# Emerging evidence-based innovative approaches to control catheter-associated urinary tract infection: a review

**DOI:** 10.3389/fcimb.2023.1134433

**Published:** 2023-07-25

**Authors:** Shobana Rajaramon, Karthi Shanmugam, Rambabu Dandela, Adline Princy Solomon

**Affiliations:** ^1^ Quorum Sensing Laboratory, Centre for Research in Infectious Diseases (CRID), School of Chemical and Biotechnology, SASTRA Deemed to be University, Thanjavur, India; ^2^ Department of Industrial and Engineering Chemistry, Institute of Chemical Technology, Bhubaneswar, Odisha, India

**Keywords:** catheter-associated urinary tract infection, surface modification, urinary catheter coatings, anti-fouling, antimicrobial, biofilm

## Abstract

Healthcare settings have dramatically advanced the latest medical devices, such as urinary catheters (UC) for infection, prevention, and control (IPC). The continuous or intermittent flow of a warm and conducive (urine) medium in the medical device, the urinary catheter, promotes the formation of biofilms and encrustations, thereby leading to the incidence of CAUTI. Additionally, the absence of an innate immune host response in and around the lumen of the catheter reduces microbial phagocytosis and drug action. Hence, the review comprehensively overviews the challenges posed by CAUTI and associated risks in patients’ morbidity and mortality. Also, detailed, up-to-date information on the various strategies that blended/tailored the surface properties of UC to have anti-fouling, biocidal, and anti-adhesive properties to provide an outlook on how they can be better managed with futuristic solutions.

## 1 Introduction

**Figure 1:**
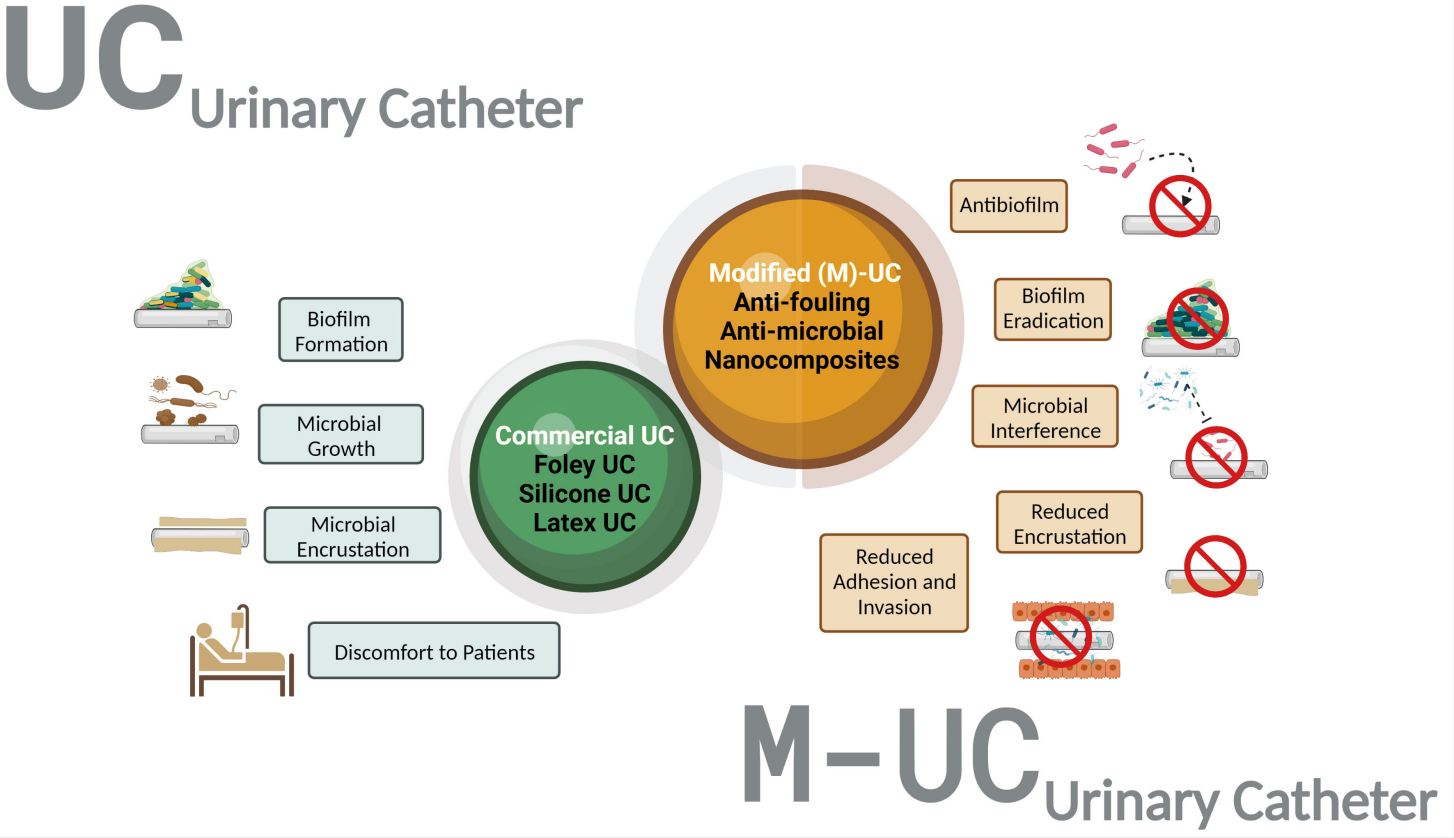
Overview of the selective advantage of Modified Urinary Catheters (M-UC) over the uncoated urinary catheter (UC). The M-UC is a tailored/blended biomaterial with various advantages over UC (prevent growth/adherence of microbes, avoid discomfort).

The advancements and developments in medical devices increase the quality and comfort of a patient’s life. However, they also pose a serious threat of acquiring Device related nosocomial infections, imparting a burden on the healthcare industry. Among various infections, the higher percentage is contributed by Catheter-Associated Urinary Tract Infections(CAUTI). Recently, many ingenious catheter coatings have been developed to prevent infection and biofilm formation on the device’s surface. This review aims to provide a comprehensive overview of various surface coatings and modifications to either prevent bacterial adherence and biofilm formation or kill the pathogen (**Figure 1**).

## 2 Urinary catheters

The urinary system is an excretion route for waste and toxic materials. Kidneys and ureters reside in the upper tract of the urinary system, where they convert liquid waste into urine and other products, whereas the bladder in the lower tract stores urine before being expelled from the body through the urethra (Hickling et al., 2015). Several risk factors, such as nerve damage and enlargement of the prostate and urethra, impair bladder function in hospitalized patients, resulting in urinary retention requiring a urinary catheter. Urinary catheters (UC) replace bladder function to drain urine in patients before, during, or after surgery and prevent urine retention in intensive care patients (Cortese et al., 2018; Feneley et al., 2015).

A UC is a long tube structured with a polymeric material that is conveniently inserted into the urethra until the urine flows through the line. It is biocompatible, with improved softness, malleability, resistance to chemicals, and smooth urine flow (Lawrence and Turner, 2005; Singha et al., 2017), and provides a short- or long-term solution to patients’ correlated medical conditions. The single-use UC is employed for males who suffer from mental disabilities or face trouble urinating. Intermittent or short-term UC is used in hospitalized patients for a maximum of 30 days and in patients under postoperative care unable to urinate. Catheterization lasting longer than 30 days is considered long-term or chronic catheterization (Donlan R., 2001). Foley catheters are latex catheters most commonly used during long-term catheterization in patients with multifactorial medical conditions, such as spinal cord injuries, multiple sclerosis, prostate enlargement, and cerebrovascular damage (Köves et al., 2017; Singha et al., 2017).

Modern medical devices have revolutionized the quality of life of patients with chronic diseases. Paradoxically, both short- and long-term catheterization have disadvantages. In the USA, approximately 15–25% of hospitalized patients (more than 30 million) use urethral and bladder catheters annually (Siddiq and Darouiche, 2012). Clinical data reveal that 10–50% of patients with non-Foley catheterization have a high incidence of catheter-associated bacteriuria (Zhang, S. et al., 2019). Monospecies cause nearly 15% of catheter-associated bacteriuria, which later develops into polymicrobial conditions, wherein the pathogens adhere to the catheters; however, the risk is minimal because they are placed in the body for a short period (Singha et al., 2017).

The European and Asian guidelines on the management of catheter-related infections list the bacterial pathogens commonly seen in short-term catheterization (*Escherichia coli*, *Proteus mirabilis*, *Klebsiella pneumonia*, *Pseudomonas aeruginosa*, *Staphylococcus epidermidis*, *Enterococcus* spp., and *Candida* spp.) (Tenke et al., 2008; Köves et al., 2017). Prolonged Foley catheterization damages urothelial cells and urethra and weakens the immune system, creating an optimal environment for bacterial adhesion, invasion, and bacteriuria, ultimately leading to CAUTI (Köves et al., 2017; Juanjuan et al., 2021; Werneburg, 2022).

## 3 Catheter-associated urinary tract infection

The National Healthcare Safety Network, managed by the Center for Disease Control [CDC], 2020 and Prevention, defines CAUTI as an infection established in a urinary catheter *in situ* for more than two days (considering device placement day as day 1) or within 48 h prior to infection onset (Roshni et al., 2013). The infection is correlated with primary symptoms, such as fever (>38.0°C), suprapubic tenderness, costovertebral angle pain or tenderness, urinary urgency, and dysuria (Fakih et al., 2022).

CAUTI is the fourth most threatening nosocomial infection worldwide (Potugari et al., 2020). In 40% of all hospital-wide infections, 80% of the cases were estimated to be CAUTI. It accounts for nearly one-third of all device-associated infections and increases morbidity and mortality in hospitalized patients (Maharjan et al., 2018; Zhang, S. et al., 2019). The annual requirement for bladder catheters in the US has increased (more than 30 million), resulting in an exponential incidence of CAUTI (Siddiq and Darouiche, 2012). Additionally, the annual cost associated with CAUTI prevention is estimated to range from $115 million to $1.82 billion (Werneburg, 2022). Thus, the global burden of CAUTI is associated with medical, social, and financial resources (Feneley et al., 2015). The primary cause of CAUTI is colonization by pathogens and their inherent ability to form biofilms. The most common pathogens associated with this infection are *P. aeruginosa, S. aureus, Enterococcus faecalis*, and *E. coli*. Other bacteria include coagulase-negative staphylococci, *S. epidermidis, K. pneumonia P. mirabilis, Proteus Vulgaris*, and *Candida albicans* (Stickler, 2014; Kart et al., 2017)

## 4 Pathogenesis of CAUTI

The pathogenesis of CAUTI begins with the entry of bacterial pathogens, followed by endoluminal or extraluminal colonization of the urinary catheter, leading to biofilm formation (Aumeran et al., 2021). Meanwhile, host defense strategies clear pathogens during voiding or intrinsic antibacterial action under normal conditions owing to the glycosaminoglycan coating on the urothelial cells (D. Zhang et al., 2004). However, the first line of host-mediated defense is neutralized under Foley catheterization because of bacterial entry and colonization of the catheter (Feneley et al., 2015).

The entry of bacteria into the urinary tract is a high-risk during catheter insertion. Once bacteria enter, they colonize the intraluminal and extraluminal surfaces of the portion of the urinary catheter inserted into the urethra (Chuang and Tambyah, 2021). Clinical data show that approximately 20% of patients suffering from CAUTI have bacterial adherence and colonization during catheter insertion (Köves et al., 2017). Several host factors, such as increased ionic strength due to the deposition of host urinary components, proteins, acidic pH, and electrolytes, lead to microbial adherence to the UC surface (Saini et al., 2017; Goda et al., 2022). In addition, host proteins cover the catheter surface and create a thin film (biofilm); this further increases bacterial adhesion to the catheter surface and/or uroepithelium, further exacerbating the formation of a thick protective layer(Djeribi et al., 2012; Chuang and Tambyah, 2021).

## 5 Rise of biofilm

The National Institutes of Health reports that approximately 80% of microbial infections and 65% of nosocomial infections are biofilm-mediated (Römling and Balsalobre, 2012). Biofilm formation on the UC surface is a phenomenon in which pathogens self-sustain to escape the host defence (Köves et al., 2017). Three-dimensional structured biofilms formed on UC are complex, with homogenous/heterogeneous sessile consortiums (Gupta et al., 2016; Azeredo et al., 2017; Costerton et al., 1999; Liu et al., 2019; Sánchez et al., 2021). This complexity is reflected in the various stages of biofilm formation, which involve reversible and irreversible binding, colonization, maturation, and dispersion (Köves et al., 2017). The initial stage of sessile microorganism attachment to a UC is usually weak and reversible and controlled by the material characteristics of the catheter, such as surface polarity, surface charges, van der Waals forces, and hydrogen bonding (Anjum et al., 2018; Alotaibi and Bukhari, 2021). However, the appendages, such as flagella, pili, and fimbriae, help them adhere to the catheter, and over time, microorganisms overcome the electrostatic repulsive forces and solvation effect that inhibit adhesion (Faustino et al., 2020). The hydrophobic and hydrophilic nature of UC allows a wide range of pathogens to form biofilms on catheters (Singha et al., 2017).

During biofilm colonization of the UC, microorganisms build a self-secreted extracellular matrix polymeric substance (EPS). EPSs are co-structured with extracellular DNA, exopolysaccharides, proteins, nucleic acids, and lipids (Liu et al., 2019). Thus, EPS acts as a scaffold built *via* secreted adhesive substances, which stabilize them to form a thick biofilm that protects pathogens from various threats. Microorganisms within the biofilm exhibit phenotypic alterations in the growth rate and production of exopolysaccharides that entrap and protect them (Donlan and William Costerton, 2002; Saini et al., 2017). In addition to increasing the concentration of intracellular signals, acyl-homoserine lactone and autoinducer peptides in gram-negative bacteria and gram-positive bacteria, respectively, initiate communication among both bacterium types to choreograph changes in the expression of genes within the microbial community and establish infection/other processes to sustain life *via* quorum sensing (QS) (Kim et al., 2012; Maharjan et al., 2018).

Once the biofilm matures on a medical device surface, it leads to failure and increases the risk of CAUTI in patients (Danese, 2002). After maturation, the biofilm tends to disperse, initiating the spread of infections downstream of the catheter (Liu et al., 2019). Dispersed cells eventually cause systemic infections, particularly in immunocompromised patients (Faustino et al., 2020). As discussed earlier, the biofilm’s threat lies in its ability to produce EPS; the matrix formed by bacteria on the urethral surface not only precludes the pathogen against the innate immune system but also contributes to antimicrobial resistance (AMR) (Stewart and William Costerton, 2001; Yassin et al., 2019). AMR is critical to understanding the involvement of biofilm because it neutralizes the effect of antimicrobial agents (Stewart and William Costerton, 2001). Several characteristics of biofilms in UCs affect antibiotic penetration, with the altered environmental conditions that favor the growth of the heterogenic cells to exhibit a resistant and persistent state (Ramadan et al., 2021).The continued race for the fast-paced development of antimicrobial agents is overcome by the ability of resistant and persistent superbugs to produce strong biofilms. Thus, the eradication of already-formed biofilms on UC surfaces in CAUTI patients is complex. Therefore, it is essential to develop a biomaterial that can efficiently control biofilm-mediated infections in medical devices (Anjum et al., 2018; Faustino et al., 2020). Although research on various biomaterials is emerging, there is also a necessity to follow the guidelines provided by the CDC, which includes the appropriate use of catheters, aseptic methods for insertion and maintenance of catheters, and catheter materials used (Centers for Disease Control and Prevention [CDC], 2020).

## 6 Urinary catheter biomaterials

The biological response to a UC depends on the surface properties of the biomaterials used. Standard biomaterials used to optimize functional characteristics include silicone, latex, polyvinyl chloride (PVC), plastic, siliconized latex, and polyurethane (PU). Microbial biofilm formation and subsequent incidence of CAUTI lead to a decreased economic value of UCs. Such challenges are overcome by modifying the structural and functional aspects of existing UCs by engineering the surfaces with potential antimicrobial/adhesive properties (Andersen and Flores-Mireles, 2019). This includes surface-engineered biomedical devices with inherent anti-fouling, biocidal, and anti-adhesive properties (Faustino et al., 2020). (Siddiq and Darouiche, 2012; Tenke et al., 2012; Ramasamy and Lee, 2016).

## 7 Anti-fouling approaches

**Figure 2:**
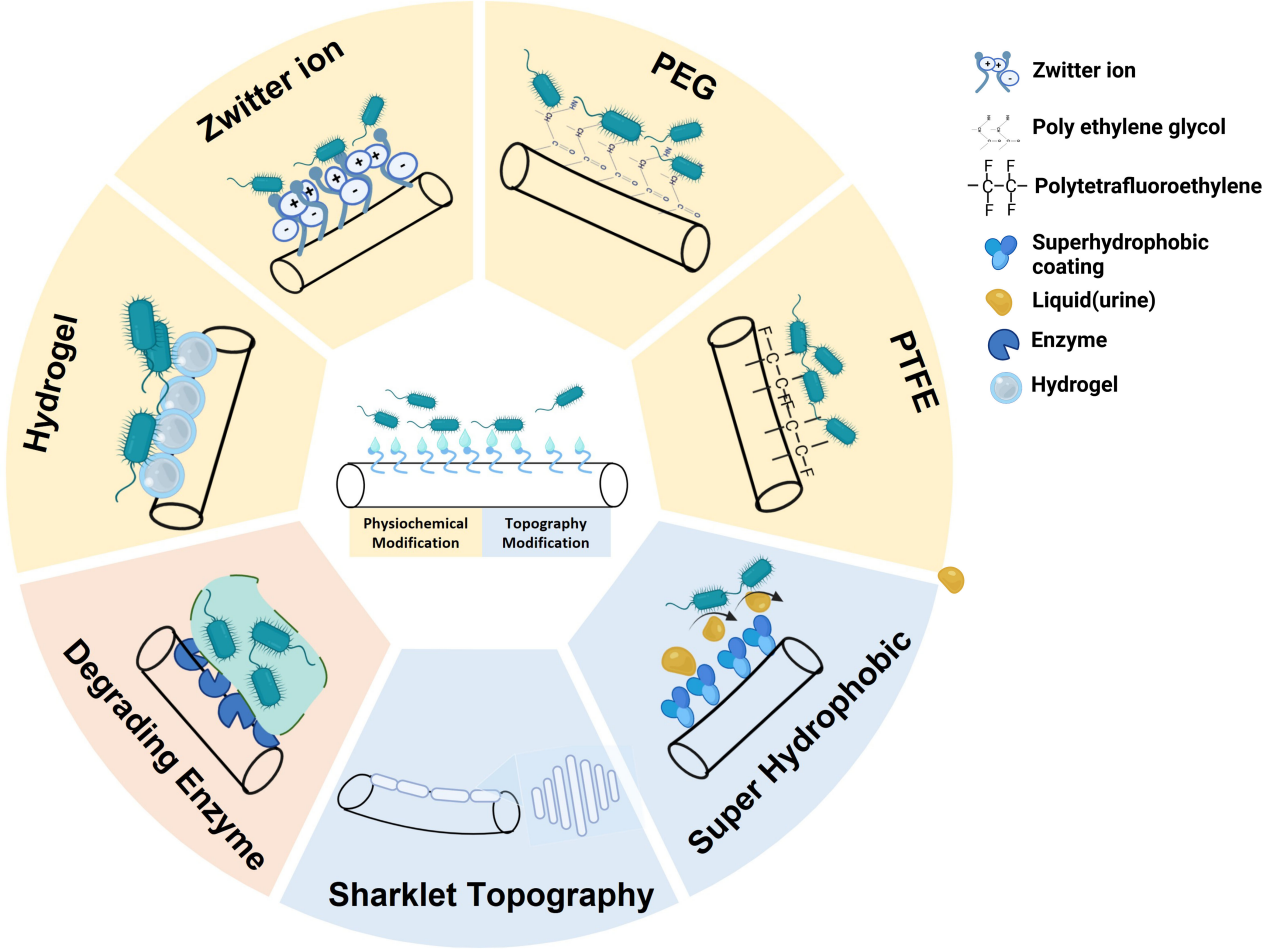
Overview of Urinary Catheter(s) (UCs) surface modification to prevent the adherence of pathogens. Top: Physiochemical modification (Hydrogel, Zwitter ions, Polyethylene glycol (PEG) and Polytetrafluoroethylene (PTFE). Bottom: Topography modification (Superhydrophobic and Sharklet topography). Left: Enzymes (Extracellular Matrix polymeric Substance (EPS) degrading enzymes). (Created using Biorender).

Anti-fouling strategies involve the surface modification of biomaterials to exhibit anti-adhesive properties that prevent microbial biofilm formation (Faustino et al., 2020). However, the selective advantage of such anti-fouling catheters is that they either prevent bio-foulant attachment or degrade them (Banerjee et al., 2011). Once the increased hydrophilic nature of the catheter is inversely proportional to microbial adherence (Desrousseaux et al., 2013). Anti-fouling catheters impart adhesion resistance owing to the functionalization of surfaces with hydrogel, polytetrafluoroethylene (PTFE) coating, and poly (ethylene glycol) (PEG) (**Table 1**, **Figure 2**). In contrast, clinical data based on studies using animal models have shown increased resistance to antibiotics associated with the long-term use of biocidal coatings.

**Table 1:** Surface modifications and their biological efficacy to control growth/biofilm on Urinary catheters.

UC Surface modification	UC considered	Active ingredient used	Methodology adopted to coat UC	Tested Pathogens	Phase of Trial	Outcomes	References
Hydrophilic	p-HEMA	1. Rifampin 2. cefixime trihydrate	Rifampin, cefixime trihydrate hydrogel coating in a combined ratio	1.*S. aureus* 2. *E. coli* 3. *P. aeruginosa*	1.Covidien Dover hydrogel coated latex catheter, USA – commercially available 2.Bardex Catheter, USA – commercially available	1. Anti-microbial (> 8 days) 2. Delayed biofilm formation	(Tarawneh et al., 2022)
	PU, PVC	1. Chitosan	Catechol functionalized Chitosan (CHI-C) hydrogel with silver nanoparticle coated on a PDA PU/PVC treated surface	*1. S. aureus* 2. *E. coli*	In research	1. Reduced bacterial adherence and biofilm formation (>20 days)	(Yang et al., 2019)
	Silicone foley catheter	1. Silver and PTFE (Ag-PTFE)	Ag-PTFE nanocomposite coating by incorporating PTFE nanoparticles into the Ag matrix	1. *S. aureus* 2. *E.coli*	BARD PTFE coated latex catheter, USA	1. Reduced biofilm and growth (>14 days)	(Zhang et al., 2019)
	Silicone foley catheter	1. Methoxylated polyethylene glycol 2. 3,4-dihydroxyphenylalanine (DOPA)	Novel silver-containing, polymer-based (mPEG-DOPA_3_) Coating	1. *E.coli* 2. *E. faecalis*, 3. *P.mirabilis*	In research	1. Reduction in biofilm formation (*in vitro*) 2. Reduction in microbial count (*in vivo*) 3. No effect on encrustation (*in vivo*)	(Tailly et al., 2021)
	PDMS strips	1. PDMS surface functionalized with zwitter ionic moieties	PDMS functionalized using the oxidation of laccase and gallic acid to trigger an enzymatic reaction of polymerization of zwitterionic sulfobetaine methacrylate monomers on the silicone catheters.	1. *S. aureus* 2. *P. aeruginosa*	In research	1. Reduction of biofilm formation (>80%). *	(Diaz Blanco et al., 2014)
Hydrophobic	Silicone catheter	1. Modification using1H,1H,2H,2H-perfluorodecanethiol using layer-by-layer deposition.	PDA coating was used as a platform attach of AgNps, followed by hydrophobic modification with 1H,1H,2H,2H-perfluorodecanethiol.	1. *E. coli* WT F1693 2. *P.mirabilias* WT F1697,	In research	1. Delayed the bacterial migration 2. Reduced biomass accumulation 3. Exhibited good biocompatibility.	(Zhang et al., 2020)
	PDMS	Trifluoropropyl	Using spray coating technique TFP was coated on PDMS	1. *P. mirabilias*		1. Reduction in bacterial attachment > 14 days. 2. Enhanced anti-biofilm activity	(Gayani et al., 2021)
	Silicon surface	Micropatterning	Three variations of sharklet micropatterned silicone surface	1. *E. coli*	In research	1. Inhibited colonisation and migration	(Reddy et al., 2011)
	PDMS elastomer	Sharklet AF™	Engineered surface microtopography based on the skin of sharks, Sharklet AF™	1. *S. aureus*		1. Delayed early biofilm formation	(Chung et al., 2007)
Enzymes	PDMS catheter	Cellobiose dehydrogenase	antimicrobial enzyme coating, produces hydrogen peroxide using oligosaccharides.	1. *S. aureus*	In research	1. Reduced viability (60%). 2. Decreased total biomass deposition on the surface	(Thallinger et al., 2016)
	PDMS catheter	Cellobiose dehydrogenase	Layer-by-layer deposition on surface with polyanions consisting of PSS and CDH, polycations consisting of novel copolymers (PTMAEMA-co-PSPE) with different sulfobetaine fractions for antifouling properties, and the addition of quaternary hydrophobic groups for contact biocide functionality.	1. *S. aureus* ATCC 10145	In research	1. Reduced the amount of biofilm development.	
	silicone catheters	Acylase and Amylase	Silicone catheter was coated with acylase and α-amylase alone and in combination using a layer-by-layer deposition technique.	1. *S. aureus* 2. *P. aeruginosa* 3. *E. coli*	In research	1. Inhibited aggregation (*in vitro*). 2. Enhanced antibiofilm activity (*in vitro*) 3. Synergistically reduced biofilm formation (70%) *in vivo*	(Ivanova et al., 2015)

### 7.1 Hydrogel-coated catheters

Advancements in hydrogel technology are effective in modifying hydrophobic catheters to more hydrophilic ones to decrease the formation of microbial biofilms on them. This is achieved using a 3D network of hydrogels made of polymers that are crosslinked, insoluble, and swellable, providing unique ice-like characteristics to improve the mechanical strength of catheters (Bahram et al., 2016; Werneburg, 2022). Interestingly, the swelling aspect of the hydrogel invariably increased hydrophilicity, providing a hydration layer for the UC to improve patient comfort and decrease microbial adherence. Meanwhile, the tissue-catheter interface reduces the encrustation and non-specific adsorption of proteins, which is a critical factor for microbial adherence (Siddiq and Darouiche, 2012; Roshni et al., 2013; Andersen and Flores-Mireles, 2019).

Although a correlation exists between the hydrophilic nature of catheters and anti-fouling properties, the real-time cell-based analysis of its potency to reduce CAUTI remains controversial, attributed to various physiochemical properties of the catheter or the types of hydrogels used (Siddiq and Darouiche, 2012). However, several success stories are coming in the future. One such clinical trial compared the efficacy of hydrogel-based UC with that of other silicone catheters in small animal models. Animals with silicone catheters had mild forms of inflammation in the urethral tissue, whereas the hydrogel catheters blocked encrustation. Likewise, in an extended study, hydrogel-based catheters showed a low level of irritation in the mucosal tissue and a subsequent decrease in bacterial adherence when compared with PTFE-coated and silicone catheters (Werneburg, 2022). In addition, catheters with the poly-2-hydroxyethyl methacrylate (p-HEMA) polymer surface loaded with rifampin (RIF) and CFX showed stable antimicrobial activity for eight days when compared to surface-modified catheters with p-HEMA alone. Furthermore, RIF and CFX enhanced the durability of the catheter employed before replacement (Tarawneh et al., 2022). Another study involved the design of a novel urethral catheter surface engineered with multiple layers using polymers, such as polydopamine (PDA) with catechol-conjugated biomolecules and loaded antibacterial agents, that demonstrated a robust effect. Additionally, hydrogels impregnated with silver nanoparticles (AgNPs) minimized bacterial adhesion. Overall, the attempt toward hydrogel-based surface engineering increased the hydrophilicity, which was expected to be stable with the desired lubrication and antimicrobial fouling effect. Stability was observed for 20 days, and both PVC and hydrogel-coated UC surfaces showed hydrated conditions. However, many additional materials require tailoring for biocompatible hydrogels as a safe and stable drug delivery system to provide a long-term effect against microorganisms that adhere to the surface of catheters (Yang et al., 2019). Hydrogel-coated catheters do offer short-term benefits of greater patient comfort and reduced microbial adherence. However, their long-term use is a concern due to the cytotoxic potential of hydrogels due to the presence of unreacted monomers, and also the physicochemical properties of the hydrogels are not clearly understood (Siddiq and Darouiche, 2012; Dai et al., 2023). This can lead to changes in the surface properties of the hydrogel and promote microbial adhesion, increasing the risk of CAUTI. Further research is needed to understand the long-term safety and efficacy of hydrogel-coated catheters fully.

### 7.2 Polytetrafluoroethylene coating

The PTFE-coated catheters (Teflon-coated catheters) were commercialized by Bard Medical (Andersen and Flores-Mireles, 2019; Zhang et al., 2021). The inherent non-sticky nature of PTFE makes it ideal for use as a material for catheter coatings. This is in accordance with a recent study where silver-PTFE (Ag-PTFE) nanocomposite-coated catheters reduced *E. coli* and *S. aureus* adherence (>55%) and biofilm coverage (>96%) when compared to uncoated commercial silicone catheters (Zhang et al., 2019). However, it cannot be stated that PTFE-coated catheters are better than commercial uncoated catheters because of the wavelet pattern that enables bacterial adherence.

### 7.3 Polyethylene glycol

Poly (ethylene glycol) and poly (ethylene oxide) (PEO) are excellent materials for catheter surface modification (Yassin et al., 2019). PEG has a high molecular weight and is well-documented as a gold-standard biocompatible material suited to coat medical devices. In addition, the anti-fouling properties of PEG are the outcome of its hydration and steric hindrance effects, which are controlled by its polymer chain length and surface packing density (Faustino et al., 2020). In a recent *in vitro* study, catheters coated with the copolymers methoxylated polyethylene glycol (mPEG) and 3,4-dihydroxyphenylalanine (DOPA) with silver cross-linking were evaluated for their efficacy against uropathogen adherence; these catheters significantly reduced the adherence of *P. mirabilis, E. faecalis*, and *E. coli*. In a rabbit model, the microbial count of *E. coli* GR 12 reduced; however, encrustation was identified (Ko et al., 2008; Tailly et al., 2021). In addition, titanium surfaces physiochemically modified using polymers, such as poly (methacrylic acid), PU acetate, and PEG, prevent protein absorption and inhibit bacterial adherence (Ramasamy and Lee, 2016).

### 7.4 Polyzwitterions coating

Polymers that possess both cationic (quaternary ammonium salt) and anionic groups (sulfonate, carboxylate, or phosphonate) in their polymeric repeating units are known as polyzwitterions. Zwitterionic polymers have excellent anti-fouling properties (Wang et al., 2022). Silicone catheters enhanced the anti-fouling properties of zwitterionic moieties when covalently modified using enzymes (laccase). The improved bioconjugate coating was evaluated for its efficacy *in vitro* under static and dynamic conditions against the pathogens *P. aeruginosa* and *S. aureus* and showed a >80% decrease in biofilm formation when compared to unmodified catheters (Diaz Blanco et al., 2014). Noteworthy data were obtained in similar studies in which silicone and latex catheters were evaluated against *P. mirabilis*. Zwitterions can either repel or prevent pathogen colonization, biofilm formation, and encrustation (Kanti et al., 2022).

### 7.5 Micropatterning of surfaces

Surface topography affects microbial adherence and subsequent biofilm formation on hydrophobic catheter surfaces (Cheng et al., 2019; Faustino et al., 2020). Several bioinspired structures have similar properties that contribute to the anti-fouling properties of the catheter surfaces (Damodaran and Sanjeeva Murthy, 2016).

#### 7.5.1 Lotus leaves-inspired superhydrophobic coating

Bioinspired superhydrophobic urinary catheters were designed using a layer-by-layer deposition technique, an innovative solution to reduce CAUTI The superhydrophobic catheters prevented uropathogenic *E. coli* WT F1693 and *P. mirabilis* WT F1697 biofilms under static and dynamic conditions and also delayed encrustation in the catheter lumen (Zhang et al., 2020). The antifouling nature of the superhydrophobic catheter was significant in comparison with other variants of silicone and silver-alloy-hydrogel catheters. Furthermore, Ag nanoparticles were endowed on the superhydrophobic surface to enhance antibacterial efficacy, improve biocompatibility, and reduce bacterial attachment. Several other studies employed a similar approach of spray coating PDMS with trifluoropropyl for catheters to provide a self-cleaning activity that decreased microbial biofilm formation over 14 days (Gayani et al., 2021).

#### 7.5.2 Sharklet topography

Sharklet AF™, a novel surface technology designed with a sharklet micropattern using a PDMS elastomer (PDMSe), possesses the inherent capacity to prevent colonization, migration, and growth of uropathogenic *E. coli* (Reddy et al., 2011). Sharklet AF™ PDMSe effectively prevented *S. aureus* biofilms for 21 days, suggesting that the topographical surface of the catheter did not show evidence of early biofilm colonization (Chung et al., 2007). However, the study must be expanded to a polymicrobial environment and requires pre-clinical evaluation to understand its safety and efficacy in the patients.

### 7.6 Matrix degrading enzymes

Exopolysaccharides and polymeric substances are the major constituents of the biofilm, providing a protective sheath to microbes. Enzyme-coated catheters have been explored for clinical use to break this protective layer.

#### 7.6.1 Cellobiose dehydrogenase

Plasma-activated urinary PDMS catheter surfaces were covalently grafted with cellobiose dehydrogenase (CDH), an antimicrobial enzyme that metabolizes oligosaccharides as electron donors to produce hydrogen peroxide in the presence of an electron acceptor (oxygen; O_2_). CDH-functionalized PDMS surfaces reduced *S. aureus* viable cells in the biofilm to >60%; these catheters are biocompatible, as they were non-toxic in mammalian cell lines (Thallinger et al., 2016). Later, an improved PDMS catheter was designed using layer-by-layer assembly in the presence of functional polymeric building blocks. The block consisted of polyanions, poly (styrene sulfonate), and CDH for antibacterial coating as the first layer. The second layer was built using polycations consisting of novel anti-fouling copolymers with zwitterionic and quaternary ammonium side groups (PTMAEMA-co-PSPE). The final layer was laid with sulfobetaine fractions and quaternary hydrophobic groups for contact biocide functionality to reduce the adherence of *S. aureus* ATCC 10145 by >60%. In addition, the combined antimicrobial coating of the catheter enhanced its killing effect (Vaterrodt et al., 2016).

## 8 Antimicrobial coatings and impregnation

**Figure 3:**
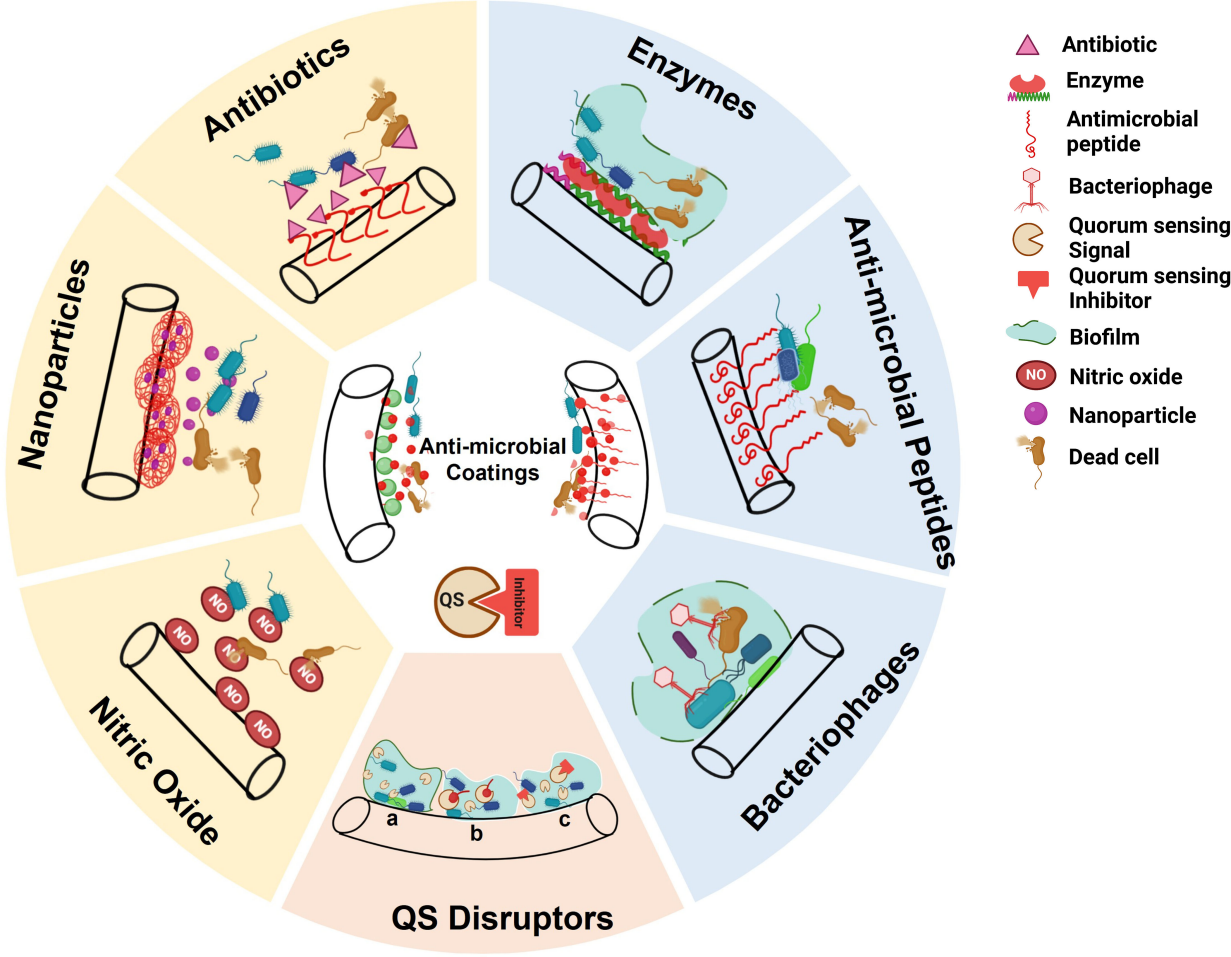
Anti-microbial coatings on Urinary Catheter(s) (UCs) to either kill the pathogens or inhibit biofilm formation. Right: Contact killing (Enzymes, Anti-microbial peptides (AMPs), Bacteriophages). Left: Release killing (Antibiotic, Nanoparticles (NPs), Nitric Oxide (NO)). Bottom: Quorum Sensing (QS) disruptors to prevent biofilm formation: a) QS mechanism b) Quorum Quenching c) QS inhibitor. (Created using Biorender).

Urinary catheter surfaces functionalized to conjugate antimicrobials, such as metal ions, antibiotics, antimicrobial peptides, bacteriophages, quorum sensing disruptors, bacterial interference, natural polymers, and bioactive molecules, have been extensively explored (**Tables 2**, **Figure 3**) (Lim et al., 2015). Such modified antimicrobial-coated catheters decrease the viability of the pathogen by inhibiting cell wall proteins and nucleic acid synthesis or blocking any specific metabolic pathway that sustains their life. Several of these have been investigated for their efficacy in controlling UTIs using *in vitro* and *in vivo* models (Roshni et al., 2013).

**Table 2:** Nanoparticles/composites and their biological efficacy to control growth/biofilm on Urinary catheters.

Type of Nanoparticle	Metal used	Mode of action	Nanocomposite	Microorganism tested	Phase of Trial	Major outcomes	References
Inorganic nanoparticles	Silver alloy coatings	Oxidative damage	Hydrogel, along with a layer of silver coating	*1. P. aeruginosa* *2. C. albicans*	Commercialised as Bardex I.C	1. Lowered the adhesion of organisms	(Ahearn et al., 2000)
			Nitrofurazone/silver alloy coated hydrogel catheter compared to PTFE catheter	1. Uropathogens		1. Excellent antimicrobial effect	(Pickard et al., 2012)
	Gold nanoparticles	Collapsing membrane potential	*Aegle marmelos* extract capped in gold nanoparticles	*1. S. aureus* *2. K.pneumonia* *3. P.aeruginosa* *4. E. faecalis*	In research	1. Inhibited the growth until 48hrs.	(Arunachalam et al., 2014)
	Silver nanoparticles	Disrupt cell wall and metabolic pathway	Spirulina platensis extract was used to synthesise SNPs, coated on catheters in combination with commercial antibiotics	*1. E.coli*	In research	1. Resisted attachment until day8 2. Exhibited 90% inhibition (2 years)	(Mala et al., 2017)
	Green Silver based nanoparticles		EPS (Kocuran) from K. rosea strain capped in SNP.	*1. S. aureus* *2. E. Coli*	In research	1. Bactericidal property 2. Inhibition of biofilm formation.	(Kumar and Sujitha, 2014)
	Copper nanoparticles	Interaction with proteins and DNA	Silver-copper (Ag-Cu) nanocomposite at various concentrations was sputtered as a film.	1. *E.coli* K12	In research	1. Showed antimicrobial effect.	(Rtimi et al., 2016)
	Zinc doped nanoparticles		Zn2+ ions doped in CuO nanoparticles were sonochemically coated.	*1. E.coli* ATCC 25922 *2. S. aureus* ATCC 29213 *3. P.mirabilis*	In research	1. Exhibited good anti-biofilm activity (24 hrs) *in vitro*.	(Shalom et al., 2017)
	Mesoporous silica nano-composite		MSNP conjugated with phenazine-1-carboxamide (PCN), a small molecule derived from K. rosea strain	*1. C. albicans*	In research	1. Anti-fungal activity	(Kanugala et al., 2019)
Organic nanoparticles	Amino- cellulose nanospheres		ACN synthesised sonochemically was functionalised on the PDMS surface	*1. E.coli*	In research	Exhibited anti-biofilm activity	(Fernandes et al., 2017)
	Sodium dodecyl sulfate nanoporous film		1,2-polybutadiene-b-polydimethylsiloxane (1,2-PB-b-PDMS) was loaded with SDS to form a nanoporous film	*1. E.coli*	In research	1. Resulted in Anti-biofilm (1 week) 2. Anti-adhesion activity (3 days)	(Li et al., 2013)

### 8.1 Metal-based approaches

#### 8.1.1 Silver alloy coating

Silver (Ag), a non-specific antimicrobial, exhibits broad-spectrum antibacterial effects at low concentrations (Zhang et al., 2019). Silver alloy coatings in catheters exist in different forms, such as silver oxide, silver alloys, and silver nanoparticles. Silver alloy releases ions that lead to oxidative DNA damage of pathogens and disrupt the cell membrane (Durán et al., 2016). In addition, silver ions activate vital enzymes that interact with thiol groups and enhance pyrimidine dimerization *via* a photodynamic approach, causing changes in the cell wall by inducing electron-dense granules (Matsumura et al., 2003). Silver ions are medically important because they are effective against a broad range of bacterial pathogens, specifically methicillin-resistant *S. aureus*. However, pathogens such as *K. pneumoniae, Enterobacter cloacae, P. mirabilis*, and *C. freundii* are emerging as silver-resistant (Estores, 2008). Catheters coated with silver oxide have no market value as they are ineffective in preventing CAUTI (Hooton et al., 2010). Several meta-analyses show that asymptomatic bacteriuria and CAUTI can be effectively reduced using Ag alloy-coated UCs (Ha and Cho, 2006; Hooton et al., 2010; Francolini et al., 2017). Researchers reviewed eight different randomized controlled trials of Ag alloy catheters, confirming their better effects than uncoated catheters; thus, they are recommended for patients at the highest risk of developing severe consequences from UTI (Donlan and William Costerton, 2002). The recommendations were contrary to other research findings; sparfloxacin (SPA)-treated urinary catheters showed better efficacy in inhibiting *E. coli* and *S. aureus* growth and biofilm formation relative to Ag-coated catheters (Kowalczuk et al., 2012). Some studies suggest that the Ag alloy-coated catheter delays the onset of infection and does not prevent the occurrence of CAUTI (Zhang et al., 2019).

The drawbacks of silver alloy catheters were addressed by embedding them in a hydrogel to provide a novel hydrogel/silver catheter to prevent CAUTI. The hydrogel/silver catheter efficiently prevented the access of microbes, including gram-positive cocci and yeasts, to the urinary tract extraluminally (Ahearn et al., 2000). A similar study with an Ag-alloy hydrogel-coated catheter was conducted in clinical settings by comparing it with a commercial catheter; a CAUTI rate reduction of 47% was observed (Davenport and Keeley, 2005; Lederer et al., 2014). The Bardex I.C. hydrogel latex Foley catheter, commercially available in the market, has its interior and exterior surfaces lined using a monolayer of Ag, which helps in reducing friction and irritation during catheterization and also provides broad-spectrum antimicrobial protection. (Singha et al., 2017) (C.R. Bard, Inc. 2008-US Patent Application Publication No. US2008/0206943 A1)

#### 8.1.2 Inorganic nanoparticles

Nanoscale materials (nanoparticles; NPs) constitute a broad spectrum of materials, including particulate substances with dimensions <100 nm (Murthy, 2007). NPs can be used as a drug delivery vehicle owing to a high surface area to volume ratio, improved pharmacokinetics and biodistribution, high solubility and stability, and decreased toxicity in comparison to conventional drug delivery systems. Moreover, the physicochemical properties of NPs can be tailored by altering their structural and functional properties to enhance their application potential in the treatment of CAUTI (Din et al., 2017).

#### 8.1.3 Gold nanoparticles

Gold nanoparticles (AuNPs) exert bactericidal activity against MDR gram-negative bacteria by collapsing membrane potential or inhibiting protein synthesis (Cui et al., 2012). A study showed that the modification of commercial PVC with methylene blue and 2 nm AuNPs upon exposure to red laser light for 4–8 min increased the photosensitivity of pathogens *S. epidermis* and *E. coli* (Noimark et al., 2012). *Aegle marmelos* leaf extract capped with AuNPs has antimicrobial activity against various biofilm-forming organisms on urinary catheters (**Table 3**) (Sánchez et al., 2021; Filipović et al., 2022).

**Table 3:** Antibiotic coatings and their biological efficacy to control growth/biofilm on Urinary catheters.

UC considered	Antibiotics	Approach used for coating UC	Tested Microorganism	Mode of killing	Phase of Trial	Major outcomes	References
Silcone catheter	Nitrofurazone	Nitrofurazone impregnated catheter	1.Uropathogens	Release - killing	Rochester Medical release-NF catheter,USA (Commercially available and later withdrawn from market)	1. Reduced Catheter-associated bacteriuria and funguria	(Stensballe, 2007)
Foley catheters		Nitrofurazone-impregnated catheter, Ag-coated silicone catheter, hydrophilic-coated catheter without an antimicrobial agent, silico-latex catheter without antimicrobial agent, silicone catheter without an antimicrobial agent.	*1. E.faecalis* *2. S.epidermis* *3. P. aeruginosa*			1. Exhibited prolonged antimicrobial durability	(Kart et al., 2017)
Silicone catheter	Chlorohexidine	Chlorohexidine along with Triclosan impregnated catheter	*1. S. aureus* *2. E. coli* *3. E.aerogenes* *4. K.pneumoniae* *5. P. mirabilis* *6. E. faecalis* *7. C. albicans*	Release - killing	In research	1. Prevented microbial colonization (20 days).	(Anjum et al., 2018)
Silicone surface		Chlorhexidine-loaded polycaprolactone nanospheres was spray coated on the surface	1. Uropathogens			1. Showed 3-fold antibacterial activity t>15 days	(Phuengkham and Nasongkla, 2015)
Silicone catheter	Gendine	Gendine was coated on a silicone catheter (GND-UC)	*1. E. coli* *2. P.aeruginosa* *3. K.pneumoniae* *4. C. albicans* *5. C. glabrata* *6. C. krusei*	Release - killing	In research	1. Exhibits 4-to 6-log reduction in biofilm (*in vitro*) 2. Reduced bacteriuria and bacterial burden (*in vivo*)	(Hachem et al., 2009)
Silicone catheter	Gentamicin	Poly(ethylene-co-vinyl acetate) and poly(ethylene oxide blends containing gentamicin were coated using the dip method	*1. P.vulgaris* *2. S.aureus* *3. S.epidermidis*	Release - killing	In research	1. Exhibited sustained drug release (7 days) 2. Exhibited anti-microbial activity (7 days)	(Cho et al., 2003)
Silicone foley catheter		Catheter was coated with poly(ethylene glycol), gentamicin sulphate and finally poly(vinyl alcohol) using the dip method	*1. E. coli* 2. *S. aureus*			1. Prevents bacterial colonization.	(Rafienia et al., 2013)
Low Density Polyethylene (LDPE) catheters	Triclosan	Triclosan was added to the LDPE at 0.10 wt.%, 0.50 wt.%, 1.00 wt.% and 1.50 wt.%.	1. *E.coli* 2. ATCC8739 *3. P.aeruginosa* ATCC 9027 4. *S.choleraesuis* ATCC 14028 *5. B. subtilis* ATCC 6633 *6. C.sporogenes* ATCC 11437 *7. E. faecalis* ATCC 29212 *8. S. aureus* ATCC 25923	Release - killing	In research	1. Imparts efficient biocidal property 2. Biofilm formation increased with decreasing triclosan. 3. Increased pH leads to encrustation and biofilm formation.	(Thomé et al., 2012)
Silicone foley catheter	Norfloxacin	EVA/PEO2kPDMS blends were used to coat the catheter surface and impregnated with norfloxacin	*1. E.coli* *2. K.pneumoniae* *3. P. vulgaris*	Release - killing	In research	1. Exhibited continuous delivery of norfloxacin (30 days)	(Park et al., 2003)
Silicone foley catheter	Ciprofloxacin	Ciprofloxacin liposome containing hydrogel was used for catheters	*1. E. coli*	Release - killing	In research	1. CAUTI was delayed (*in vivo*)	(Pugach et al., 1999)
Silicone	Ciprofloxacin with azithromycin	Combination of azithromycin and ciprofloxacin coating was prepared using a solvent-based method	1. *P. aeruginosa* PAO1	Release - killing		1. Antimicrobial effect for a prolonged period of time. 2. Prevention of biofilm formation and stable shelf-life for one year.	(Saini et al., 2016)
polyurethane stents	Ciprofloxacin with N-acetylcysteine	Ciprofloxacin in combination with N-acetylcysteine was coated using the dip method	*1. S.aureus* *2. S.epidermidis* *3. E.coli* *4. K.pneumoniae* *5. P.aeruginosa* *6. P.vulgaris* *7. P.rettgeri* *8. C.freundii* *9. S. marcescens.*	Release - killing		1. Dose-dependent Inhibition of microbial adherence. 2. Broad spectrum, prolonged antimicrobial effect	(El-Rehewy et al., 2009)
Foley catheter	Nitric oxide	A piece was catheter was impregnated with nitric oxide using a chamber	*1. E. coli*	Release - killing	In research	1. Prevented biofilm formation	(Regev-Shoshani et al., 2010)
Silicone catheter	RK1 (RWKRWWRRKK), RK2 (RKKRWWRRKK)	Covalently tethering of RK1 and RK2 *via* allyl glycidyl ether polymer brush on PDMS surface	*1. E.coli* *2. S.aureus* *3. C.albicans*	Contact-Killing	In research	1. Showed antimicrobial effect 2. Prevented biofilm 3. Non-toxic to host cells	(Li et al., 2014)
PDMS surface	CWR11	Synthetic CWR11was immobilised on PDMS support by Covalent immobilisation *via* intermediate crosslinking using PDA film	*1. E.coli* ATCC 8739 2. *S.aureus* ATCC 6538 3. *P.aeruginosa* PAO1	Contact-Killing	In research	1. Potent bactericidal properties 2. Potent salt-resistant properties	(Lim et al., 2013)
Silicone Foley catheters	Cys Lasio-III	CysLasio-III was immobilised on commercial catheter using an AGE brush platform	1. *E. coli* ATCC8739 2. 2. *P. aeruginosa* 3. ATCC9027 4. 3. *S. aureus* 5. ATCC6538 6. 4. *E. faecalis* 7. ATCC29212	Contact-Killing	In research	1. Exhibited antimicrobial and anti-adhesive properties. 2. Stable for 4 days in urine.	(Mishra et al., 2014)
	39APmC32, 65APm2833, 72APm5211	Phages were studied alone and as a cocktail	*1. 1. P. mirabilis*	Contact-Killing	In research	1. Possess an anti-biofilm agent. 2. Stable under adverse milieu conditions.	(Maszewska et al., 2018)
Bladder model	Siphovirus (Isf-Pm1) and Myovirus (Isf-Pm2)	Phage cocktail was prepared using Isf-Pm1 and Isf-Pm2	*1. 1. P. mirabilis* ATCC 7002	Contact-Killing	In research	1. Achieved 4-log reduction in biofilm formation 2. Downregulation of adhesion-associated genes.	(Mirzaei et al., 2022)
Silicone and latex catheters	Chrysophanol	The catheter was coated by dipping in the chrysophanol-AgNPs solution containing long-chain dodecyl methacrylate.	*1. *1. *P. aeruginosa* *2. *PAO1 *3. *2. *E. coli* (MTCC 443)	Quorum sensing disruptors	In research	1. Showed 9-fold anti-adhesion and anti-biofouling effects. 2. Reduced biofilm formation	(Prateeksha et al., 2021)
Silicon catheter	Chitosan	The chitosan extracted from shells of crab *P. sanguinolentus* was coated as in solution form using a dip coating technique	*1. *1. *S.epidermidis* *2. *(RP62A) (ATCC 35984) *3. *2. *C.albicans* *4. *(ATCC 90028)		In research	1. Downregulated the virulence genes (*bhp* and *agrAC*) in *S.epidermis* (*ume6* and *hyr1*) in *C. albicans*	(Rubini et al., 2021)
latex catheter	Salicyl acrylate	Polyurethane acrylate polymer composed of salicyl acrylate was co-cured to make films. It was coated on catheters using the dip coating method.	1. *P. aeruginosa* 2. *E. coli*		In research	1. anti-biofilm property, under simulated physiological urine flow simulation.	(Chifiriuc et al., 2012)

#### 8.1.4 Silver nanoparticles

Silver-based nanomaterials have gained more attention than AuNPs as well-established (Makabenta et al., 2021) metal antimicrobials that disrupt both bacterial cell walls and metabolic pathways (Sim et al., 2018; Sánchez et al., 2021). Commercial UCs coated with self-polymerized polydopamine act as active platforms for the deposition of silver nanoparticles in situ, preventing biofilm formation by gram-positive bacterial pathogens^36^. Interestingly, AgNPs exert antibacterial/biofilm and biofilm activities on coagulase-negative *S. aureus* (Thomas et al., 2015). Foley catheters were coated with AuNPs along with various combinations of antibiotics (amikacin (6.25 µg/mL) and nitrofurantoin (31.25 µg/mL) to observe the combinatorial effect under *in vitro* and *in vivo* conditions. These catheters significantly controlled the colonization of microbes until the 14^th^ day of observation in the mouse model. In addition, the functionalized catheter anti-adherence activity was evaluated two years after storing it aseptically and was proven to be efficient in controlling microbial biofilm >90% (**Table 3**). Thus, the impregnation of UC with AuNPs and antibiotics is promising for preventing biofilm formation (Mala et al., 2017).

#### 8.1.5 Green silver-based nanoparticles

The green synthesis of NPs using biological extracts of lower to higher organisms is safer, non-toxic, biocompatible, and cost-effective (Iravani, 2014; Pal et al., 2019; Sánchez et al., 2021). In this pipeline, green synthesis of AgNPs using the *Kocuria rosea* strain BS-1 showed antimicrobial and antifouling activity against *S. aureus* and *E. coli.* The data was extrapolated toward functionalizing the same (Kocuran-functionalized AgNPs) in urinary silicone catheters, which showed the same response in controlling microbial adherence (Kumar and Sujitha, 2014). Similar studies were conducted in varnishing green AgNPs synthesized using *Pistacia lentiscus* (mastic), which also prevented bacterial colonization. The AgNPs synthesized using pomegranate grind extract-coated catheters inhibited bacterial colonization for >72 h by antibiotic-resistant clinical gram-positive (*S. epidermidis* and *S. aureus*) and gram-negative (*E. coli, K. pneumoniae, P. mirabilis*, and *P. aeruginosa*) bacteria but were more active against gram-negative bacteria(Goda et al., 2022). *C*arissa *carandas* leaf extract-capped silver nanoparticles (AgNPs) coated catheters with commercial antibiotics (ciprofloxacin: 50 mcg, trimethoprim: 30 mcg, and gentamycin: 30 mcg) show antimicrobial and antifouling activities (**Table 3**) (Rahuman et al., 2021).

#### 8.1.6 Copper-based nanoparticles

Copper nanoparticles affect bacterial cell functions in various ways, adhering to the gram-negative bacterial cell wall *via* electrostatic force, denaturing the intracellular protein, and further interacting with phosphorus and sulfur-containing molecules, such as DNA (Mahmoodi et al., 2018). A notable added value was observed when hybrid bimetal Cu-Ag NP-coated catheters reduced the viable cell count of *E. coli* (Andersen and Flores-Mireles, 2019).

#### 8.1.7 Zinc-doped copper nanoparticles

Zn-doped CuO (Zn_0.12_Cu_0.88_O) nanoparticle-coated catheters catheterized in a rabbit model displayed biocompatibility, antibiofilm effects and low cytotoxicity. The nanoparticles, analyzed for seven days, effectively prevented CAUTI. Taken together, these data emphasize the therapeutic potential (antifouling) of Zn-doped CuO nanocomposites (Shalom et al., 2017).

#### 8.1.8 Mesoporous silica-based nanocomposite

Mesoporous silica nanoparticles (MSNPs) are a class of Food and Drug Administration (FDA)-approved nano-drug delivery systems with the selective advantage of being stable and having customizable pore size, increased surface area, and pore volume, enabling a variety of chemical modifications to improve its functional properties for diagnosis and therapy(Gao et al., 2020). In addition, MSNPs act as both drug carriers and imaging modalities (Farjadian et al., 2019). MSNPs functionalized with phenazine-1-carboxamide (PCN) (PCN-MSNPs) were evaluated against Candida spp. and *S. aureus* and exhibited a 4-fold increase in antibiofilm activity. These data were incomparable to those of pure PCN. Mechanistic studies showed that PCN-induced intracellular reactive oxygen species accumulation, reduction in membrane permeability and total ergosterol content, and disruption of ionic homeostasis with the release of Na^+^, K^+^, and Ca^2+^ leakage led to the death of *C. albicans* and *S. aureus* (Kanugala et al., 2019). Furthermore, a detailed investigation of its efficacy in suitable *in vivo* models is warranted before clinical application (Farjadian et al., 2019; Gao et al., 2020).

#### 8.1.9 Other inorganic nanoparticles

Nanoparticles of tungsten, titanium, sulfur, and hydroxyapatite also show antimicrobial and antifouling activity against uropathogens. Tungsten nanoparticle (W-NP)-coated catheters show bactericidal effects at low concentrations in both clinical and standard drug-resistant pathogenic *E. coli* (Syed et al., 2010). The green synthesis of sulfur nanoparticles (SNPs) in the presence of *Catharanthus roseus* leaf extract exhibited antibacterial activity against uropathogens, either alone or in combination with selected antibiotics, such as amoxicillin and trimethoprim (Paralikar et al., 2019). In this pipeline, hydroxyapatite (HA) nanoparticle-coated urethral catheters were tested in rabbit models, and the formation of biofilm on the luminal surface of the catheters was significantly reduced compared to the control until the catheterization period (5–7 days) (Evliyaoğlu et al., 2011).

#### 8.1.10 Organic nanoparticles

Polymeric NPs are drug carriers that release antimicrobial agents, bacteriostatic peptides, alkyl pyrimidines, or quaternary ammonium compounds to enable the contact killing of pathogens(Ramasamy and Lee, 2016). A nanoporous polymer film prepared from self-polymerized 1,2-polybutadiene-b-polydimethylsiloxane (1,2-PB-b-PDMS) block copolymers *via* chemical cross-linking of the 1,2-PB block and sodium dodecyl sulfate blocked *E. coli* attachment and biofilm for one week. The durability of the activity over seven days was due to the tailoring of the morphological features of the nanoporous polymer films (Li et al., 2013). As a continued effort, researchers devised a catheter material, PDMS, with a known antibacterial component, amino cellulose nanospheres, using epoxy/amine grafting chemistry, which reduced the total biomass in the *E. coli* biofilms when compared with the naked silicone catheter (**Table 3**) (Fernandes et al., 2017).

### 8.2 Antibiotic coating

#### 8.2.1 Nitrofurazone

Nitrofurazone, a nitrofuran derivative, is a broad-spectrum antibiotic used to treat UTIs (Lee et al., 2004). It reduces reactive intermediates by releasing nitric oxide (NO), which interferes with ribosomes, DNA, and the cell wall to inhibit bacterial replication, growth, and biofilm formation (Siddiq and Darouiche, 2012; Thompson et al., 2016). Nitrofurazone-coated catheters were tested against a wide spectrum of bacterial pathogens and were found to inhibit the adherence of *E. coli* and *E. faecalis* for 3–5 days(Stensballe, 2007; Desai et al., 2010; Kanti et al., 2022). In addition, several other comparative studies showed that the nitrofurazone-impregnated silicone catheter reduced the viable count of *E. faecalis* and inhibited *S. epidermidis* and *P. aeruginosa* biofilms (**Table 3**) (Kart et al., 2017; Al-Qahtani et al., 2019). However, a nitrofurazone-coated catheter is a promising catheter for preventing bacterial adherence and biofilm; the one that was in commercial use (Rochester Medical Release-NF catheter, USA) was withdrawn from the market as it created discomfort in patients (Zhang et al., 2021). Later, it was listed as prohibited by the FDA because it caused tumors in the animal model subjects (Singha et al., 2017).

#### 8.2.2 Chlorhexidine

Chlorhexidine (N, N‴′1,6-Hexanediylbis[N′-(4-chlorophenyl) (imidodicarbonimidic diamide) is a di-cationic bisbiguanide with broad antibacterial activity (Jones, 1997). To date, research has shown that chlorhexidine is bacteriostatic at low concentrations and vice-versa (bactericidal) in a wide range of gram-positive and gram-negative pathogens (Francolini et al., 2017). The UC is coated with chlorhexidine using either spray or dip coating methods and has potential in appropriate *in vitro* models that mimic the urinary tract, either alone or in combination with other antimicrobial agents such as triclosan (Kanti et al., 2022). The data showed that chlorhexidine- and triclosan-coated catheters could synergistically prevent the colonization of a wide range of bacterial pathogens for >20 days (**Table 2**)(Anjum et al., 2018). In 2015, polycaprolactone nanospheres loaded with chlorhexidine were spray-coated on silicone catheters to provide a sustained release of chlorohexidine compared to bulk polymers and were effective for 15 days (Phuengkham and Nasongkla, 2015; Singha et al., 2017).

#### 8.2.3 Gendine

Gendine is an antiseptic dye (gentian violet and chlorhexidine; GND-UC) with broad-spectrum activity against drug-resistant microbes that cause UTIs (Siddiq and Darouiche, 2012). Comparative studies on using GND-UC to other silver hydrogel-coated Foley catheters and uncoated catheters under *in vitro* conditions suggested that GND-UC is dominant and exhibits better effects against MDR *E. coli, P. aeruginosa*, ESBL *K. pneumoniae*, VRE, methicillin-resistant *S. aureus*, and Candida spp. The extended *in vivo* study showed similar observations, with a significant decrease in biofilm formation compared to silver-alloy-coated and uncoated catheters (Hachem et al., 2009).

#### 8.2.4 Gentamicin

Gentamicin belongs to the class of aminoglycosides known to exert bactericidal effects on a broad range of pathogens, excluding *Streptococcus* and *Enterococcus* spp. They exert their action *via* interacting with the protein synthesizing machinery, specifically the A-site of the 30S ribosome (Krause et al., 2016). To apply gentamicin as a therapy to treat CAUTI, the UCs were coated with this antibiotic with appropriate delivery vehicles known as gentamicin-containing poly (ethylene-co-vinyl acetate) (EVA) and EVA/poly (ethylene oxide) for local and sustained release for a prolonged period of seven days against *P. vulgaris, S. aureu*s and *S. epidermis*. *In vivo*, experiments recommend its application to treat short-term catheterization, as it can deteriorate biofilms for 3–5 days (Cho et al., 2003; Ha and Cho, 2006; Rafienia et al., 2013; Roshni et al., 2013; Andersen and Flores-Mireles, 2019). However, studies have shown that when the gentamicin delivery vehicle is changed to PEG, there is a significant change in the release profile, which is extended to 12 days (Rafienia et al., 2013). Although significant results have been obtained, gentamicin-releasing catheters have limitations because gentamicin is a hydrophilic antibiotic known for its rapid release from the carrier; thus, it may not be suitable for short-term catheterization (Ha and Cho, 2006).

#### 8.2.5 Triclosan

Triclosan (TCS) is a synthetic lipid-soluble antimicrobial agent, also known as 5-chloro-2-(2,4-dichloro phenoxy) phenol. It blocks a critical enzyme, enoyl-acyl carrier protein (ACP) reductase (FabI), a critical enzyme that affects the growth of microbes and is involved in fatty acid biosynthesis. In addition, it was observed that the same TCS might also show non-specific actions of targeting multiple pathways and destabilizing membranes (Cadieux et al., 2009; Dhende et al., 2012). However, it is harmless to humans because we lack ENR enzymes (Dhillon et al., 2015). For more than 40 years, it has been used as a disinfectant and preservative in hospitals. Later, it was proposed to have application potential to treat CAUTI *via* coating in UCs (Yueh and Tukey, 2016). Researchers have conducted detailed investigations of the antimicrobial efficacy of low-density polyethylene (LDPE) catheters with various concentrations of TCS. The addition of TCS (0.5 wt.%) is critical to achieving the minimum inhibitory concentration against multiple uropathogens (**Table 3**) (Thomé et al., 2012). Several studies have demonstrated the limitation of TCS as pathogens overexpress enoyl reductase or changes in cellular permeability impact its non-functionality for them to develop resistance^112^. Another study claimed that TCS induces efflux pump overexpression, resulting in high-level resistance in *P. aeruginosa* (Chuanchuen et al., 2001). Another report suggested that overexpression of the enzyme Fab I by 3–5 fold decreased *S. aureus* sensitivity against TCS, as mutations were mapped in the gene *FabI* of resistant strains (Fan et al., 2002). In the year, 2016 the FDA banned TCS in soap products and allowed 1% TCS in toothpaste, mouthwash, and hand sanitizer. In the subsequent year, the European Union banned TCS from all human hygiene biocidal products (Weatherly and Gosse, 2017).

#### 8.2.6 Norfloxacin

The UC was coated with norfloxacin, a hydrophobic wide-spectrum synthetic antibiotic coated on UC to prevent CAUTI (Park et al., 2003; Ha and Cho, 2006). Norfloxacin was coated on the surface of the catheters with EVA/PEO2kPDMS (poly (ethylene-co-vinyl acetate; EVA), and an amphiphilic multiblock copolymer (poly (ethylene oxide) and poly (dimethyl siloxane); PEO2kPDMS). After coating, norfloxacin release kinetics were established to provide better treatments for patients with long-term catheterization. Experiments showed that EVA/PEO2kPDMS blends containing norfloxacin inhibited selective pathogens (*E. coli, K. pneumoniae*, and *P. vulgaris*) over 10 days of treatment. Combinatorial studies of the same blends with norfloxacin and other antibiotics, such as ciprofloxacin and azithromycin, proved to be effective against microbial biofilms; however, further exploration at the preclinical level is warranted (Park et al., 2003; Saini et al., 2016).

#### 8.2.7 Ciprofloxacin

Ciprofloxacin (CFX) is an antibacterial agent used to treat a range of infections, including skin, ophthalmic, bone, respiratory, and urinary tract infections (Vidyavathi and Srividya, 2018). A previous study reported that pathogens treated with sub-MIC concentrations of CFX decreased their biofilm, lowering their hydrophobicity (El-Rehewy et al., 2009). Its efficacy over biofilm inhibition expanded its application potential in catheters, and the CFX-coated catheters were evaluated in rabbit models and showed inhibition of *E. coli* on catheter biofilms (Pugach et al., 1999). Furthermore, a combination of CIP with azithromycin (AZM) showed better efficacy in inhibiting *P. aeruginosa* (PA01) growth for 30 days, suggesting its stability, shelf life, and future application for the treatment of long-term CAUTI. Further *in vivo* analysis in the murine model suggests that AZM-CFX-coated catheters were efficient in tackling CAUTI in comparison to the uncoated catheters, which showed persistent colonization of pathogens as well as the dispersion in urine to spread infection further (Saini et al., 2017). In another study, the effects of CFX and N-acetylcysteine-coated catheters, alone and in combination, were evaluated for their antimicrobial adherence activity against various pathogens (**Table 2**). Ciprofloxacin/N-acetylcysteine-impregnated catheters resulted in the highest inhibitory effect on microbial adherence when compared with controls (85.5%–100%)(El-Rehewy et al., 2009).

#### 8.2.8 Sparfloxacin

Sparfloxacin (SPA)-coated latex catheters were tested against *E. coli* and *S. aureus* using broth and agar diffusion methods. The antifouling effect of SPA was compared with that of silver-coated and uncoated catheters. It was observed to significantly reduce the colonization of *E. coli* and *S. aureus* compared to both the silver-coated and untreated catheters (Kowalczuk et al., 2012). Furthermore, the same group developed an antimicrobial urological catheter surface modified with a thin film of heparin (HP) deposited with SPA, which was stable to provide long-term antibacterial protection (Kowalczuk et al., 2010).

#### 8.2.9 Other antibiotics

Although several antibiotics are being explored for use in the treatment of CAUTI, research on other antibiotics is ongoing. The efficacy of the bladder catheter coated with antibiotics (minocycline and rifampin) showed a decrease in the rate of catheter-associated bacteriuria over two weeks compared to that of the control (Darouiche et al., 1999). Meanwhile, the efficacy of third generation cephalosporins, ceftazidime, and ceftriaxone-coated catheters to prevent *P. aeruginosa* biofilm formation has been investigated. The antibiotics ceftazidime and ceftriaxone delay biofilm formation for more than a week and are recommended for short-term catheterization but not for long-term catheterization (>28 days) (Ghanwate et al. 2014).

### 8.3 Nitric oxide

Nitric oxide (NO) is a free-radical gas that is hydrophobic in nature and significantly affects innate immunity. NO is highly diffusive and has a short half-life in a physiological milieu. It NO has a short half-life in a physiological environment and quick diffusive property *via* biological liquids, thereby binding to DNA, proteins, and lipids to inhibit or kill pathogens. (Regev-Shoshani et al., 2010; Schairer et al., 2012). The inherent biological activity of NO is extrapolated to its application potential in impregnating Foley urinary catheters. The impregnated Foley UC releases NO into the urine and is stable for two weeks in various clinical models. Thus, it prevented bacterial colonization on the external and luminal surfaces and was able to eradicate up to 10^4^ CFU/ml of *E. coli* in the surrounding media under static and dynamic conditions (Regev-Shoshani et al., 2010). Research has also shown the influence of pH on NO activity. Therefore, subsequent experiments were conducted in varying urine pH, and results showed that the release of NO was different, which may affect the response to CAUTI treatment (Andersen and Flores-Mireles, 2019).

### 8.4 Antimicrobial peptides

Antimicrobial peptides (AMPs) are short, cationic molecules that are part of the innate immune system of many species and exhibit effective antimicrobial activity. The unique ability of AMPs to affect the viability of bacteria is higher than that for conventional antibiotics owing to their amphipathic nature (Camesano et al., 2015). Thus, the amphipathic nature of AMP either kills the bacteria *via* membrane disruption or enters cells by interacting with their biomolecules vital for intracellular functions without membrane disruption (Benfield and Henriques, 2020). The therapeutic potential of AMPs has paved the way for their exploration as coating candidates to curb CAUTI. Two novel AMP candidates, RK1 and RK2, were immobilized on PDMS and UC surfaces *via* an allyl glycidyl ether (AGE) polymer brush interlayer (**Table 3**). The AMP-coated catheters showed antimicrobial action against *E. coli, S. aureus*, and *C. albicans* without exerting any toxicity on smooth muscle cells. The combinatorial effect of the AGE polymer brush and AMPs showed a synergistic interaction, repelling cell adhesion and biofilm formation (Li et al., 2014).

Furthermore, the same group of researchers tested the effect of an AMP candidate, CWR11 (arginine-tryptophan-rich peptide coated on a PDMS-functionalized catheter surface). Notably, it affected a wide range of pathogens *via* disruption of the membrane without exerting any toxicity (Lim et al., 2013). Later, in 2014, this was improved by adopting a bioinspired PDA-based coating for AMP grafting. CWR11 was tethered onto catheter surfaces by depositing a thin film of PDA onto the PDMS surface to enhance the attachment of CWR11. The CWR11-deposited UC showed bactericidal properties for 21 days against both gram-positive and gram-negative bacterial pathogens without affecting host immunity and uroepithelial cells. The net effect of the experimental trial is a proof-of-concept that demonstrates the potential role of PDA–CWR11-functionalized catheters in combating CAUTIs (Lim et al., 2015).

Meanwhile, a hydrophilic polymer coating (AMP coating on PU) with anti-adherence properties was developed and examined *in vitro*. The AMP was labeled at the C-terminus (RRWRIVVIRVRRC) with cysteine, and PU tethered with AMP-coated UC decreased free-living cells and sessile cells by >70% and >99%, respectively. The study was extended to a suitable *in vivo* CAUTI mouse model and observed to be biocompatible with the host cells and, in parallel, inhibited the viability of cells when compared to an uncoated surface. The AMP-brush coating also showed host bladder epithelial fibroblast cells in cell-based assays (Yu et al., 2017).

Another potent AMP, lasioglossin-III (Lasio-III) peptide, was chemically modified with a cysteine at the N-terminal, also known as CysLasio-III. CysLasio-III was immobilized on the UC with the support of a PEG spacer and site-directed coupling at the N-terminus of the peptide. Immobilization on the UC was enabled using sulfhydryl coupling to direct the proper orientation of CysLasio-III, as it influences the biological activity against gram-positive and gram-negative pathogens. The biological activity of CysLasio-III was examined and was found to be effective against *E. coli* and *E. faecalis* biofilms for four days. This is the first proof-of-concept to demonstrate the biocompatibility and application potential of site-directed sulfhydryl immobilization of CysLasio-III on UC ability against CAUTI-relevant pathogens (Mishra et al., 2014).

An improved AMP-impregnated PEG–polycaprolactone (PCL)-coated UC showed controlled release with antimicrobial properties. Once the ratios of the PEG and PEG–PCL copolymers were varied, they resulted in different morphologies and indirectly affected the AMP release profiles. The formulation with 10% (w/w) PEG–PCL in PCL exerted a controlled AMP release for >2 weeks with a moderate initial burst release. The optimized coating was further evaluated on a silicone catheter, and it outperformed in reducing biofilm formation than the other silver-based antimicrobial catheters with antimicrobial performance and sustainability lasting less than a week. However, the potential therapeutic value of AMPs in UC is still challenged by various suboptimal coating strategies using non-specific immobilization chemistry, changing the orientation of AMPs and associated toxicities in the host cells (Mishra et al., 2014). Research based on AMPs is at an early stage and needs to be evaluated in *in-vivo* conditions, focusing on peptide degradation and chemical reactions.

### 8.5 Bacteriophages

In the era of increasing AMR, alternate therapy with bacteriophages competes with other antimicrobial therapies as they confer a selective advantage. The advantage of phage therapy is its ability to act against microbes and prevent them from acquiring AMR (Maszewska et al., 2018).Several studies have reported that the challenge can be overcome with the “bacteriophage cocktail”. Detailed research on the effect of various combinations of 13 phages against the free/sessile form of 50 uropathogenic *P. mirabilis* was conducted. The cocktails of phages 39APmC32, 65APm2833, and 72APm5211 inhibited bacterial biofilms. In 2022, the same group identified a potent cocktail using lytic phages (Isf-Pm1 and Isf-Pm2) to inhibit *P. mirabilis* biofilms. Notably, phages deficient in lysogenization can impact the 4-log reduction of *P. mirabilis* biofilms on silicone catheters. RT-PCR data revealed the downregulation of genes associated with QS and adhesion (Mirzaei et al., 2022).

An infection-responsive surface-coated UC was designed to release a therapeutic dose of bacteriophage in response to elevated urinary pH and delay catheter blockage. The design is dual-layered, wherein the lower layer is the hydrogel “reservoir” immobilized with a bacteriophage cocktail. The upper layer holds the pH-responsive polymer (poly (methyl methacrylate-co-methacrylic acid) (EUDRAGIT^®^S 100)) that acts as a trigger layer to release the appropriate dose of phages in response to urine pH changes. The dual-layered UC coatings were stable in the presence or absence of pathogens deficient in urease. In addition, naked-eye visualization showed a drastic clearance of the crystalline biofilm (Milo et al., 2017). Thus, phage therapy is considered an alternative to prevent biofilm formation and blockage in UC and is a better solution to the issues faced in clinical settings.

### 8.6 Quorum sensing disruptors

Microbial biofilms are driven by bacterial communication known as quorum sensing, which has gained attention for overcoming antimicrobial resistance. Anti-QS agents act as barriers to biofilm formation by interrupting inter-/intra-species communication. These agents can either be quorum-quenching enzymes that degrade signalling molecules or quorum-sensing inhibitors that block signalling molecules and auto-inducers (Rémy et al., 2018; Majik et al., 2020).

### 8.7 Quorum-sensing inhibitors

Quorum-sensing inhibitors are highly specific to target QS regulators that do not interfere with the metabolic processes of bacteria but inhibit microbial pathogenicity without developing resistance (Soto, 2014). Researchers have examined the effect of known QS inhibitors, such as p-nitro phenyl glycerin and tannic acid, against *P. mirabilis*, and the data showed significant inhibition of biofilm growth. In search for various natural biofilm inhibitors, 3-methyl-2(5H)-furanone (furans), 2´-hydroxycinnamic acid(phenyl-acyl), was found to marginally inhibit biofilm formed by *K.pneumoniae* on urethral catheters by acting as an antagonist against, *N*-hexanoyl-homoserine lactone (C6-AHL) (Cadavid and Echeverri, 2019). 2,5-dimethyl-4-hydroxy-3(2H)-furanone (DMHF), an aromatic compound found in berries and pineapple, was found to be effective against *Candida tropicalis*, isolated from foley urinary catheters. The DMHF-coated catheter inhibited biofilm formation completely in comparison to silicone, latex and foley catheters (Devadas et al., 2019). In addition, nanoparticle have been explored for modulating QS to reduce bacterial pathogenicity and suppress bacterial adhesion and colonization (Jones et al., 2009). Similar studies were extended to a natural anthraquinone, chrysophanol, isolated from an endolichenic fungus (ELF), talaromyces wortmannin MN243726, which was used to functionalize NPs (CP-AgNPs) for anti-QS and antifouling therapies. The anti-adherence/anti-fouling properties of the CP-AgNP-coated UC surfaces were effective at inhibiting the growth of *P. aeruginosa* PAO1 and *E. coli* in static/dynamic conditions. A comparative evaluation of CP-AgNP-coated latex/silicon with the citrate-capped AgNPs and UC surfaces showed a several-fold increase in the same effect observed above. This observation was significant because they were also able to influence the downregulation of pathogenicity without exerting toxicity on the host cells. The study was extended *in vivo*, where CP-AgNPs showed a strong influence in preventing biofouling and provided excellent protection to patients with UC-associated UTIs (Prateeksha et al., 2021).

#### 8.7.1 Quorum-quenching enzymes

Quorum-sensing interaction throws light upon Quorum Quenching, allowing researchers to disrupt bacterial communication and biofilm formation using various modes of action. One possible way to interrupt QS is signal inactivation by enzymatic degradation or modification. This led to the discovery of quorum-quenching enzymes, such as acylase and amylase (Fetzner, 2015; Murugayah and Gerth, 2019). Multilayer UC coatings with either acylase or amylase suppressed the biofilm formation of *P. aeruginosa* and *S. aureus.* In a recent study, hybrid nanocoatings with QS-signal-degrading acylase enhanced biofilm inhibition of clinically relevant bacterial pathogens in both mono and mixed species in static and dynamic conditions. Moreover, quorum quenching, and matrix-degrading enzymes offset the growth of biofilms for up to seven days in an *in vivo* animal model (Ivanova et al., 2015). In addition, lactonases produced by *Bacillus thuringiensis* (AiiA), archaeon *Sulfolobus solfataricus*(SsoPox) hydrolysis the AHL’s secreted by the pathogen to protect themselves could also be tested as a potential catheter coating to prevent degrade QS signal and inhibit biofilm formation(Rémy et al., 2016; Bzdrenga et al., 2017).

### 8.8 Bacterial interference

CAUTI-associated microbes compete differentially with each other in the process of nutrient acquisition and surface colonization. During this competition for survival, they antagonize or interfere with other bacteria, affecting their colonization and invasion of host defense (Falagas et al., 2008). The natural mechanism has been extrapolated to the treatment of CAUTI in clinical settings. UC pre-exposure to *E. coli* 83972 antagonized the colonization of uropathogens (*Providencia stuartii*, uropathogenic lactose-negative *E. coli*, and *C. albicans*) (Trautner et al., 2003). However, the broad therapeutic interference warrants *in-vivo* validation.

### 8.9 Natural polymers and bioactive materials

Various naturally occurring polymers and bioactive molecules provide eco-friendly solutions for catheter-associated infections (Rubini et al., 2019). One such natural polymer is chitosan, a marine polysaccharide isolated from the shell chitin of crustaceans (eg. marine crab *Portunus sanguinolentus*). Chitosan-based UCs are hydrophilic, with enhanced broad-spectrum activity against microbes. They eradicated the pre-formed biofilms and downregulated other virulence factors, such as slime production and QS-regulated genes (*agrAC, bhp*) in *S. epidermis* and transition in the morphogenic switch (yeast to hyphal) in *C. albicans* (Rubini et al., 2021).

Salicylic acid is a bioactive molecule that plays a major role in polymeric coating, which is coated onto a UC *via* polymer (PU). Such coatings on UCs favored the sustained release of salicylic acid and prolonged its effect against microbial biofilm formation of *P. aeruginosa* and *E. coli.* In another study, the inner lumen of UCs coated with salicylic acid-releasing polymer reduced *E. coli* biofilms for five days under physiological conditions. Essential oil nano-biosystem pellicles prepared using *Rosmarinus officinalis* inhibited the adherence ability and development of biofilms on the catheter surface by *C. albicans* and *C. tropicalis* clinical strains (Nowatzki et al., 2012). In summary, naturally occurring biomolecules have shown antibacterial and antifouling activity under *in vitro* conditions; however, they must be tested in *in vivo* CAUTI models to validate their efficacy.

## 9 Current challenges in design

It is evident from the compiled research data above that tremendous efforts are being made across the globe by various research groups to find a phenomenal solution against CAUTI in the form of urinary catheter coatings while taking various elements into account. However, very few anti-fouling strategies or antimicrobial coatings have reached the clinical trial stage or the commercial market owing to the gap between *in vitro* and *in vivo* assays in the laboratory environment and actual human anatomy. The foremost step in an *in vitro* assay is the growth of bacterial cultures in laboratory media, such as artificial urine media, Muller Hinton agar, and Tryptic Soy Agar; however, they failed to recapitulate the catheterized bladder environment (Sarigul et al., 2019). Additionally, the flow pattern of urine, real stress conditions, and compound stresses in the bladder were not considered under *in vitro* conditions (Xiong et al., 2021).

Another concern is the concentration of O_2_ required for bacterial growth in urine culture. Numerous *in vitro* studies have been conducted in shaking environments, as they supply O_2_ to enhance bacterial growth. The level of O_2_ absorbed into the system is higher than the level of O_2_ that can be observed in patient/healthy urine samples. This implies that the outcomes differ because *in vitro* research circumstances do not replicate those of *in vivo* studies(Giannakopoulos et al., 1997; Beebout et al., 2019).

Although murine and rabbit models remain valuable in the laboratory to test various urinary catheter coatings and drugs, they fail to accurately mimic human bladder conditions. Human and mouse bladders vary in anatomical features and the expression of biomarkers on the epithelial surface. The human urothelium has 5–7 layers of cells due to the presence of intermediate cells, whereas the mouse urothelium has 3–4 layers of cells. This creates differences in the microenvironment and variations in cytokeratin profiles (Laguna et al., 2006; Murray et al., 2021). It is imperative to study CAUTI and various treatment modalities using small animal models and cell cultures; however, its limitations must be considered.

## 10 Conclusion and future outlooks

This review describes the morbidity and prevalence of CAUTIs as well as recent developments in anti-adhesive or antimicrobial catheters employing a variety of therapeutic modalities. Despite several advancements in catheter coatings, CAUTI remains a cause of nosocomial infections across the globe, and the dawn of antibiotic resistance and its effect on antibiotic treatment efficacy is alarming.

Going forward, researchers must look into new approaches that primarily prevent the adherence of bacteria to the catheter surface, for which physiochemistry or the surface topography of the catheter can be modified. Second, preventing biofilm formation would aid in the fight against multidrug resistance, as it is practically impossible to eradicate biofilms once they are formed due to the polymicrobial environment and conferred antibiotic resistance. This can be achieved by interrupting the quorum sensing system using quorum sensing inhibitors, more specifically, by blocking the efflux pumps critical for biofilm formation or degrading the signaling molecules using quorum quenching enzymes. This is an alternate approach to overcome antibiotic resistance, as it neither imposes survival stress on the microorganisms nor induces the development of antibiotic resistance. Furthermore, the synergistic effects of drugs can be explored to identify an antibiotic resistance breaker using drug cocktails in combination with adjuvants in the form of nanocomposites or nanocarriers. The use of nanomaterials as a drug-loaded system on the catheter coating would be an added advantage, as it can be exploited to meet our needs. Other alternative strategies to combat antibiotic resistance include multi-mechanism approaches, antimicrobial peptides, phage therapy, and the use of natural bioactive molecules.

## Author contributions

Study conception design and project administration: SR and AS; Original draft manuscript preparation, writing: SR; Reviewing and editing: SR, KS, RD and AS. All authors contributed to the article and approved the submitted version.

## References

[B1] Ahearn D. G. Grace D. T. Jennings M. J. Borazjani R. N. Boles K. J. Rose L. J.. (2000). Effects of hydrogel/silver coatings on *in vitro* adhesion to catheters of bacteria associated with urinary tract infections. Curr. Microbiol. 41 (2), 120–125. doi: 10.1007/s002840010105 10856378

[B2] Alotaibi G. F. Bukhari M. A. (2021). Factors influencing bacterial biofilm formation and development. Am J Biomed Sci & Res. 12 (6), AJBSR.MS.ID.001820. doi: 10.34297/AJBSR.2021.12.001820

[B3] Al-Qahtani M. Abeer S. Ghufran J. Sara A. (2019). Efficacy of anti-microbial catheters in preventing catheter associated urinary tract infections in hospitalized patients: A review on recent updates. J. Infection Public Health 12 (6), 760–766. doi: 10.1016/j.jiph.2019.09.009 31628048

[B4] Andersen M. J. Flores-Mireles A. L. (2019). Urinary catheter coating modifications: the race against catheter-associated infections. Coatings 10 (1), 23. doi: 10.3390/coatings10010023

[B5] Anjum S. Surabhi S. Lepoittevin B. Philippe R. Manoj P. Bhuvanesh G. (2018). Biomodification strategies for the development of antimicrobial urinary catheters: overview and advances. Global Challenges 2 (1), 1700068. doi: 10.1002/gch2.201700068 31565299 PMC6607219

[B6] Arunachalam K. Sathesh K. A. Arunachalam A. M. Subashini K (2014). One step green synthesis of phytochemicals mediated gold nanoparticles from Aegle marmales for the prevention of urinary catheter infection. Int J Pharm Pharm Sci 6 (1), 700-706.

[B7] Aumeran C. Mottet-Auselo B. Forestier C. Nana P.-A. Hennequin C. Robin Frédéric. (2021). A prospective study on the pathogenesis of catheter-associated bacteriuria in critically ill patients. BMC Microbiol. 21 (1), 86. doi: 10.1186/s12866-021-02147-9 33752594 PMC7983228

[B8] Azeredo J. Azevedo N. F. Briandet R. Cerca N. Coenye T. Costa A. R.. (2017). Critical review on biofilm methods. Crit. Rev. Microbiol. 43 (3), 313–351. doi: 10.1080/1040841X.2016.1208146 27868469

[B9] Bahram M. Mohseni N. Moghtader M. (2016). “An introduction to hydrogels and some recent applications,” in Emerging Concepts in Analysis and Applications of Hydrogels ((IntechOpen Limited: London, UK), pp. 9–39.).

[B10] Banerjee I. Pangule R. C. Kane R. S. (2011). Antifouling coatings: recent developments in the design of surfaces that prevent fouling by proteins, bacteria, and marine organisms. Advanced Materials 23 (6), 690–718. doi: 10.1002/adma.201001215 20886559

[B11] Beebout C. J. Eberly A. R. Werby S. H. Reasoner S. A. Brannon J. R. De S.. (2019). Respiratory heterogeneity shapes biofilm formation and host colonization in uropathogenic *Escherichia coli* . MBio 10 (2), e02400-18. doi: 10.1128/mBio.02400-18 30940709 PMC6445943

[B12] Benfield AurélieH. Henriques SóniaT. (2020). Mode-of-action of antimicrobial peptides: membrane disruption vs. Intracellular mechanisms. Front. Med. Technol. 2. doi: 10.3389/fmedt.2020.610997 PMC8757789 35047892

[B13] Bzdrenga J. Daudé D. Rémy B. Jacquet P. Plener L. Elias M.. (2017). Biotechnological applications of quorum quenching enzymes. Chemico-Biological Interact. 267, 104–115. doi: 10.1016/j.cbi.2016.05.028 27223408

[B14] Cadavid E. Echeverri F. (2019). The search for natural inhibitors of biofilm formation and the activity of the Autoinductor C6-AHL in Klebsiella Pneumoniae ATCC 13884. Biomolecules 9 (2), 49. doi: 10.3390/biom9020049 30704099 PMC6406709

[B15] Cadieux P. Wignall G. Carriveau R. (2009). “Introduction to biofilms in urology,” in Biomaterials and Tissue Engineering in Urology (Elsevier Woodhead Publishing: Cambridge, UK.), 3–41.

[B16] Camesano T. A. Alexander T. Lozeau L. (2015). Development of antimicrobial peptides for catheter-related bloodstream infectionprevention. Antimicrobial Resistance Infection Control 4 (S1), I4. doi: 10.1186/2047-2994-4-S1-I4

[B17] Centers for Disease Control and Prevention (CDC), 2020. CDC, A. W. (2020). Centers for disease control and prevention.

[B18] Cheng Y. Feng G. Moraru C. I. (2019). Micro- and Nanotopography sensitive bacterial attachment mechanisms: a review. Front. Microbiol. 10. doi: 10.3389/fmicb.2019.00191 PMC6393346 30846973

[B19] Chifiriuc C. Grumezescu V. Grumezescu A. M. Saviuc C. Lazăr V. Andronescu E. (2012). Hybrid magnetite Nanoparticles/Rosmarinus officinalis essential oil nanobiosystem with antibiofilm activity. Nanoscale Res. Lett. 7 (1), 209. doi: 10.1186/1556-276X-7-209 22490675 PMC3368737

[B20] Cho Y. W. Park J. H. Kim S. H. Cho Y.-H. Choi J. M. Shin H. J.. (2003). Gentamicin-releasing urethral catheter for short-term catheterization. J. Biomaterials Science Polymer Edition 14 (9), 963–972. doi: 10.1163/156856203322381447 14661873

[B21] Chuanchuen R. Beinlich K. Hoang T. T. Becher A. Karkhoff-Schweizer R. R. Schweizer H. P. (2001). Cross-resistance between triclosan and antibiotics in *Pseudomonas aeruginosa* is mediated by multidrug efflux pumps: exposure of a susceptible mutant strain to triclosan selects NfxB mutants overexpressing MexCD-OprJ. Antimicrobial Agents Chemotherapy 45 (2), 428–432. doi: 10.1128/AAC.45.2.428-432.2001 11158736 PMC90308

[B22] Chuang L. Tambyah P. A. (2021). Catheter-associated urinary tract infection. J. Infection Chemotherapy 27 (10), 1400–1406. doi: 10.1016/j.jiac.2021.07.022 34362659

[B23] Chung K. K. Schumacher J. F. Sampson E. M. Burne R. A. Antonelli P. J. Brennan A. B. (2007). Impact of engineered surface microtopography on biofilm formation of *Staphylococcus aureus* . Biointerphases 2 (2), 89–94. doi: 10.1116/1.2751405 20408641

[B24] Cortese Y. J. Wagner V. E. Tierney M. Devine D. Fogarty A. (2018). “Review of catheter-associated urinary tract infections and *in vitro* urinary tract models”. 2018 J. Healthcare Eng. 2018, 1–16. doi: 10.1155/2018/2986742 PMC6204192 30405898

[B25] Costerton J. W. Stewart P. S. Greenberg E. P. (1999). Bacterial biofilms: a common cause of persistent infections. Science 284 (5418), 1318–1322. doi: 10.1126/science.284.5418.1318 10334980

[B26] Cui Y. Zhao Y. Tian Y. Zhang W. Lü X. Jiang X. (2012). The molecular mechanism of action of bactericidal gold nanoparticles on escherichia coli. Biomaterials 33 (7), 2327–2333. doi: 10.1016/j.biomaterials.2011.11.057 22182745

[B27] Dai S. Gao Y. Duan L. (2023). Recent advances in hydrogel coatings for urinary catheters. J. Appl. Polym Sci. 140 (14), e53701. doi: 10.1002/app.53701

[B28] Damodaran V. B. Sanjeeva Murthy N. (2016). Bio-inspired strategies for designing antifouling biomaterials. Biomaterials Res. 20 (1), 18. doi: 10.1186/s40824-016-0064-4 PMC4913429 27326371

[B29] Danese P. N. (2002). Antibiofilm approaches. Chem. Biol. 9 (8), 873–880. doi: 10.1016/S1074-5521(02)00192-8 12204686

[B30] Darouiche R. O. Smith J. A. Hanna H. Dhabuwala C. B. Steiner M. S. Babaian R. J.. (1999). Efficacy of antimicrobial-impregnated bladder catheters in reducing catheter-associated bacteriuria: A prospective, randomized, multicenter clinical trial. Urology 54 (6), 976–981. doi: 10.1016/S0090-4295(99)00288-5 10604693

[B31] Davenport K. Keeley F. X. (2005). Evidence for the use of silver-alloy-coated urethral catheters. J. Hosp. Infection 60 (4), 298–303. doi: 10.1016/j.jhin.2005.01.026 15936115

[B32] Desai D. G. Liao K. S. Cevallos M. E. Trautner B. W. (2010). Silver or nitrofurazone impregnation of urinary catheters has a minimal effect on uropathogen adherence. J. Urol. 184 (6), 2565–2571. doi: 10.1016/j.juro.2010.07.036 21030042 PMC2991122

[B33] Desrousseaux C. Sautou V. Descamps S. Traoré O. (2013). Modification of the surfaces of medical devices to prevent microbial adhesion and biofilm formation. J. Hosp. Infection 85 (2), 87–93. doi: 10.1016/j.jhin.2013.06.015 24007718

[B34] Devadas S. M. Nayak U. Y. Narayan R. Hande M. H. Ballal M. (2019). 2,5-dimethyl-4-hydroxy-3(2H)-furanone as an anti-biofilm agent against non-candida albicans candida species. Mycopathologia 184 (3), 403–411. doi: 10.1007/s11046-019-00341-y 31187338

[B35] Dhende V. P. Hardin I. R. Locklin J. (2012). “Durable antimicrobial textiles: types, finishes and applications,” in Understanding and improving the Durability of Textiles (Elsevier: Cambridge), 145–173.

[B36] Dhillon G. Kaur S. Pulicharla R. Brar S. Cledón M. Verma M.. (2015). Triclosan: current status, occurrence, environmental risks and bioaccumulation potential. Int. J. Environ. Res. Public Health 12 (5), 5657–5684. doi: 10.3390/ijerph120505657 26006133 PMC4454990

[B37] Diaz Blanco C. Ortner A. Dimitrov R. Navarro A. Mendoza E. Tzanov T. (2014). Building an antifouling zwitterionic coating on urinary catheters using an enzymatically triggered bottom-up approach. ACS Appl. Materials Interfaces 6 (14), 11385–11393. doi: 10.1021/am501961b 24955478

[B38] Din F. ud Aman W. Ullah I. Qureshi O. S. Mustapha O. Shafique S.. (2017). Effective use of nanocarriers as drug delivery systems for the treatment of selected tumors. Int. J. Nanomedicine 12, 7291–7309. doi: 10.2147/IJN.S146315 29042776 PMC5634382

[B39] Djeribi R. Bouchloukh W. Jouenne T. Menaa B. (2012). Characterization of bacterial biofilms formed on urinary catheters. Am. J. Infection Control 40 (9), 854–859. doi: 10.1016/j.ajic.2011.10.009 22325732

[B40] Donlan R. (2001). Biofilms and device-associated infections. Emerging Infect. Dis. 7 (2), 277–281. doi: 10.3201/eid0702.010226 PMC2631701 11294723

[B41] Donlan R. M. William Costerton J. (2002). Biofilms: survival mechanisms of clinically relevant microorganisms. Clin. Microbiol. Rev. 15 (2), 167–193. doi: 10.1128/CMR.15.2.167-193.2002 11932229 PMC118068

[B42] Durán N. Durán M. de Jesus M. B. Seabra A. B. Fávaro W. J. Nakazato G. (2016). Silver nanoparticles: a new view on mechanistic aspects on antimicrobial activity. Nanomedicine: Nanotechnology Biol. Med. 12 (3), 789–799. doi: 10.1016/j.nano.2015.11.016 26724539

[B43] El-Rehewy M. S. K. Ali El-Feky M. Hassan M. A. Abolella H. A. Abolyosr A. Abd El-Baky R. M.. (2009). *In* v*itro* efficacy of ureteral catheters impregnated with ciprofloxacin, N-acetylcysteine and their combinations on microbial adherence. Clin. Med. Urol. 3, CMU.S3367. doi: 10.4137/CMU.S3367

[B44] Estores I. M. (2008). Silver hydrogel urinary catheters: evaluation of safety and efficacy in single patient with chronic spinal cord injury. J. Rehabil. Res. Dev. 45 (1), 135–140. doi: 10.1682/JRRD.2006.12.0154 18566932

[B45] Evliyaoğlu Yalçın Kobaner M. Çelebi H. Yelsel Kazım Doğan Aydın (2011). The efficacy of a novel antibacterial hydroxyapatite nanoparticle-coated indwelling urinary catheter in preventing biofilm formation and catheter-associated urinary tract infection in rabbits. Urological Res. 39 (6), 443–449. doi: 10.1007/s00240-011-0379-5 21484419

[B46] Fakih M. G. Bufalino A. Sturm L. Huang R.-H. Ottenbacher A. Saake K.. (2022). Coronavirus disease 2019 (COVID-19) pandemic, central-line–associated bloodstream infection (CLABSI), and catheter-associated urinary tract infection (CAUTI): the urgent need to refocus on hardwiring prevention efforts. Infection Control Hosp. Epidemiol. 43 (1), 26–31. doi: 10.1017/ice.2021.70 PMC8007950 33602361

[B47] Falagas M. E. Rafailidis P. I. Makris G. C. (2008). Bacterial interference for the prevention and treatment of infections. Int. J. Antimicrobial Agents 31 (6), 518–522. doi: 10.1016/j.ijantimicag.2008.01.024 18359612

[B48] Fan F. Yan K. Wallis N. G. Reed S. Moore T. D. Rittenhouse S. F.. (2002). Defining and combating the mechanisms of triclosan resistance in clinical isolates of *Staphylococcus aureus* . Antimicrobial Agents Chemotherapy 46 (11), 3343–3347. doi: 10.1128/AAC.46.11.3343-3347.2002 12384334 PMC128739

[B49] Farjadian F. Roointan A. Mohammadi-Samani S. Hosseini M. (2019). Mesoporous silica nanoparticles: synthesis, pharmaceutical applications, biodistribution, and biosafety assessment. Chem. Eng. J. 359, 684–705. doi: 10.1016/j.cej.2018.11.156

[B50] Faustino CéliaM.C. Lemos S. M.C. Monge N. Ribeiro I. A.C. (2020). A scope at antifouling strategies to prevent catheter-associated infections. Adv. Colloid Interface Sci. 284, 102230. doi: 10.1016/j.cis.2020.102230 32961420

[B51] Feneley R. C.L. Hopley I. B. Wells P. N.T. (2015). Urinary catheters: history, current status, adverse events and research agenda. J. Med. Eng. Technol. 39 (8), 459–470. doi: 10.3109/03091902.2015.1085600 26383168 PMC4673556

[B52] Fernandes M. M. Ivanova K. Francesko A. Mendoza E. Tzanov T. (2017). Immobilization of antimicrobial core-shell nanospheres onto silicone for prevention of *Escherichia coli* biofilm formation. Process Biochem. 59, 116–122. doi: 10.1016/j.procbio.2016.09.011

[B53] Fetzner S. (2015). Quorum quenching enzymes. J. Biotechnol. 201, 2–14. doi: 10.1016/j.jbiotec.2014.09.001 25220028

[B54] Filipović N. Tomić N. Kuzmanović M. Stevanović M. M. (2022). Nanoparticles. potential for use to prevent infections. In: Soria, F., Rako, D., de Graaf, P. (eds) Urinary Stents. (Cham:Springer). doi: 10.1007/978-3-031-04484-7_26

[B55] Francolini I. Vuotto C. Piozzi A. Donelli G. (2017). Antifouling and antimicrobial biomaterials: an overview. APMIS 125 (4), 392–417. doi: 10.1111/apm.12675 28407425

[B56] Gao Y. Gao D. Shen J. Wang Q. (2020). A review of mesoporous silica nanoparticle delivery systems in chemo-based combination cancer therapies. Front. Chem. 8. doi: 10.3389/fchem.2020.598722 PMC7732422 33330389

[B57] Gayani B. Dilhari A. Kottegoda N. Ratnaweera D. R. Weerasekera M. M. (2021). Reduced crystalline biofilm formation on superhydrophobic silicone urinary catheter materials. ACS Omega 6 (17), 11488–11496. doi: 10.1021/acsomega.1c00560 34056304 PMC8154006

[B58] Ghanwate N. A. Thakare P. V. Bhise P. R. Swapnil T. (2014). Prevention of biofilm formation in urinary catheters by treatment with antibiofilm agents. Int. J. Sci. Res 3, 714–717.

[B59] Giannakopoulos X. Evangelou A. Kalfakakou V. Grammeniatis E. Papandropoulos I. Charalambopoulos K. (1997). Human bladder urine oxygen content: implications for urinary tract diseases. Int. Urol. Nephrol. 29 (4), 393–401. doi: 10.1007/BF02551103 9405994

[B60] Goda R. M. El-Baz A. M. Khalaf E. M. Alharbi N. K. Elkhooly T. A. Shohayeb M. M. (2022). Combating bacterial biofilm formation in urinary catheter by green silver nanoparticle. Antibiotics 11 (4), 495. doi: 10.3390/antibiotics11040495 35453246 PMC9032029

[B61] Gupta P. Sarkar S. Das B. Bhattacharjee S. Tribedi P. (2016). Biofilm, pathogenesis and prevention—a journey to break the wall: A review. Arch. Microbiol. 198 (1), 1–15. doi: 10.1007/s00203-015-1148-6 26377585

[B62] Ha U.S. Cho Y.-H. (2006). Catheter-associated urinary tract infections: new aspects of novel urinary catheters. Int. J. Antimicrobial Agents 28 (6), 485–490. doi: 10.1016/j.ijantimicag.2006.08.020 17045784

[B63] Hachem R. Reitzel R. Borne A. Jiang Y. Tinkey P. Uthamanthil R.. (2009). Novel antiseptic urinary catheters for prevention of urinary tract infections: correlation of *in vivo* and *in vitro* test results. Antimicrobial Agents Chemotherapy 53 (12), 5145–5149. doi: 10.1128/AAC.00718-09 19805562 PMC2786341

[B64] Hickling D. R. Sun T.-T. Wu X.-R. (2015). Anatomy and physiology of the urinary tract: relation to host defense and microbial infection. Microbiol. Spectr. 3 (4), 1–25. doi: 10.1128/microbiolspec.UTI-0016-2012 PMC4566164 26350322

[B65] Hooton T. M. Bradley S. F. Cardenas D. D. Colgan R. Geerlings S. E. Rice J. C.. (2010). Diagnosis, prevention, and treatment of catheter-associated urinary tract infection in adults: 2009 international clinical practice guidelines from the infectious diseases society of America. Clin. Infect. Dis. 50 (5), 625–663. doi: 10.1086/650482 20175247

[B66] Iravani S. (2014). Bacteria in nanoparticle synthesis: current status and future prospects. Int. Scholarly Res. Notices, 2014 1–18. doi: 10.1155/2014/359316 PMC4897565 27355054

[B67] Ivanova K. Fernandes M. M. Francesko A. Mendoza E. Guezguez J. Burnet M.. (2015). Quorum-quenching and matrix-degrading enzymes in multilayer coatings synergistically prevent bacterial biofilm formation on urinary catheters. ACS Appl. Materials Interfaces 7 (49), 27066–27077. doi: 10.1021/acsami.5b09489 26593217

[B68] Jones C. G. (1997). Chlorhexidine: is it still the gold standard? Periodontology 2000 15 (1), 55–62. doi: 10.1111/j.1600-0757.1997.tb00105.x 9643233

[B69] Jones S. M. Dang T. T. Martinuzzi R. (2009). Use of quorum sensing antagonists to deter the formation of crystalline proteus mirabilis biofilms. Int. J. Antimicrobial Agents 34 (4), 360–364. doi: 10.1016/j.ijantimicag.2009.06.011 19619987

[B70] Juanjuan Du TianTian Z. Yue D. Lili W. Ping Xu Xu H. (2021). Analysis of etiology and risk factors of catheter-associated urinary tract infection in critically ill patients and research on corresponding prevention and nursing measures. Appl. Bionics Biomechanics 2021, 1–7. doi: 10.1155/2021/8436344 PMC8712177 34966446

[B71] Kanti S.P.Y. Csóka Ildikó Jójárt-Laczkovich O. Adalbert Lívia (2022). Recent advances in antimicrobial coatings and material modification strategies for preventing urinary catheter-associated complications. Biomedicines 10 (10), 2580. doi: 10.3390/biomedicines10102580 36289841 PMC9599887

[B72] Kanugala S. Jinka S. Puvvada N. Banerjee R. Ganesh Kumar C. (2019). Phenazine-1-carboxamide functionalized mesoporous silica nanoparticles as antimicrobial coatings on silicone urethral catheters. Sci. Rep. 9 (1), 6198. doi: 10.1038/s41598-019-42722-9 30996286 PMC6470230

[B73] Kart D. Kustimur AyşeS. Sağıroğlu M. Kalkancı Ayşe (2017). Evaluation of antimicrobial durability and anti-biofilm effects in urinary catheters against enterococcus faecalis clinical isolates and reference strains. Balkan Med. J. 34 (6), 546–552. doi: 10.4274/balkanmedj.2016.1853 29215338 PMC5785660

[B74] Kim J. Park H.-D. Chung S. (2012). Microfluidic approaches to bacterial biofilm formation. Molecules 17 (8), 9818–9834. doi: 10.3390/molecules17089818 22895027 PMC6268732

[B75] Ko R. Cadieux P. A. Dalsin J. L. Lee B. P. Elwood C. N. Razvi H. (2008). *First prize:* novel Uropathogen-resistant coatings inspired by marine mussels. J. Endourology 22 (6), 1153–1160. doi: 10.1089/end.2008.0049 18484883

[B76] Köves Béla Magyar András Tenke P. (2017). Spectrum and antibiotic resistance of catheter-associated urinary tract infections. GMS Infect. Dis. 5, Doc06. doi: 10.3205/id000032 30671328 PMC6301742

[B77] Kowalczuk D. Ginalska Grażyna Golus J. (2010). Characterization of the developed antimicrobial urological catheters. Int. J. Pharmaceutics 402 (1–2), 175–183. doi: 10.1016/j.ijpharm.2010.10.014 20951780

[B78] Kowalczuk D. Ginalska Grażyna Piersiak T. Miazga-Karska Małgorzata (2012). Prevention of biofilm formation on urinary catheters: comparison of the Sparfloxacin-treated long-term antimicrobial catheters with silver-coated ones. J. Biomed. Materials Res. Part B: Appl. Biomaterials 100B (7), 1874–1882. doi: 10.1002/jbm.b.32755 22903649

[B79] Krause K. M. Serio A. W. Kane T. R. Connolly L. E. (2016). Aminoglycosides: an overview. Cold Spring Harbor Perspect. Med. 6 (6), a027029. doi: 10.1101/cshperspect.a027029 PMC4888811 27252397

[B80] Kumar C.G. Sujitha P. (2014). Green synthesis of kocuran-functionalized silver glyconanoparticles for use as antibiofilm coatings on silicone urethral catheters. Nanotechnology 25 (32), 325101. doi: 10.1088/0957-4484/25/32/325101 25060660

[B81] Laguna P. Smedts F. Nordling Jörgen Horn T. Bouchelouche K. Hopman A.. (2006). Keratin expression profiling of transitional epithelium in the painful bladder syndrome/interstitial cystitis. Am. J. Clin. Pathol. 125 (1), 105–110. doi: 10.1309/W342BWMDMDDBCTVH 16482998

[B82] Lawrence E. L. Turner I. G. (2005). Materials for urinary catheters: A review of their history and development in the UK. Med. Eng. Phys. 27 (6), 443–453. doi: 10.1016/j.medengphy.2004.12.013 15990061

[B83] Lederer J. W. Jarvis W. R. Thomas L. Ritterm J. (2014). Multicenter cohort study to assess the impact of a silver-alloy and hydrogel-coated urinary catheter on symptomatic catheter-associated urinary tract infections. J Wound Ostomy Continence Nurs. 41 (5), 473–480. doi: 10.1097/WON.0000000000000056 PMC4165476 24922561

[B84] Lee S.-J. Kim S. W. Cho Y.-H. Shin W.-S. Lee S. E. Kim C.-S.. (2004). A comparative multicentre study on the incidence of catheter-associated urinary tract infection between nitrofurazone-coated and silicone catheters. Int. J. Antimicrobial Agents 24, 65–69. doi: 10.1016/j.ijantimicag.2004.02.013 15364311

[B85] Li X. Li P. Saravanan R. Basu A. Mishra B. Lim S. H.. (2014). Antimicrobial functionalization of silicone surfaces with engineered short peptides having broad spectrum antimicrobial and salt-resistant properties. Acta Biomaterialia 10 (1), 258–266. doi: 10.1016/j.actbio.2013.09.009 24056098

[B86] Li Li Molin S. Yang L. Ndoni S. (2013). Sodium dodecyl sulfate (SDS)-loaded nanoporous polymer as anti-biofilm surface coating material. Int. J. Mol. Sci. 14 (2), 3050–3064. doi: 10.3390/ijms14023050 23377015 PMC3588030

[B87] Lim K. Chua R. R. Y. Ho B. Tambyah P. A. Hadinoto K. Leong S. Su J. (2015). Development of a catheter functionalized by a polydopamine peptide coating with antimicrobial and antibiofilm properties. Acta Biomaterialia 15, 127–138. doi: 10.1016/j.actbio.2014.12.015 25541344

[B88] Lim K. Chua R. R. Y. Saravanan R. Basu A. Mishra B. Tambyah P. A.. (2013). Immobilization studies of an engineered arginine–tryptophan-rich peptide on a silicone surface with antimicrobial and antibiofilm activity. ACS Appl. Materials Interfaces 5 (13), 6412–6422. doi: 10.1021/am401629p 23758173

[B89] Liu H. Shukla S. Vera-González N. Tharmalingam N. Mylonakis E. Fuchs B. B.. (2019). Auranofin releasing antibacterial and antibiofilm polyurethane intravascular catheter coatings. Front. Cell. Infection Microbiol. 9. doi: 10.3389/fcimb.2019.00037 PMC6403144 30873389

[B90] Maharjan G. Khadka P. Shilpakar G. S. Chapagain G. Dhungana G. R. (2018). Catheter-associated urinary tract infection and obstinate biofilm producers. Can. J. Infect. Dis. Med. Microbiol. 2018, 1–7. doi: 10.1155/2018/7624857 PMC6129315 30224941

[B91] Mahmoodi S. Elmi A. Nezhadi S. H. (2018). Copper nanoparticles as antibacterial agents. J. Mol. Pharmaceutics Organic Process Res. 06 (01). doi: 10.4172/2329-9053.1000140

[B92] Majik M. S. Gawas U. B. Mandrekar V. K. (2020). Next generation quorum sensing inhibitors: accounts on structure activity relationship studies and biological activities. Bioorganic Medicinal Chem. 28 (21), 115728. doi: 10.1016/j.bmc.2020.115728 33065436

[B93] Makabenta J. M. V. Nabawy A. Li C.-H. Schmidt-Malan S. Patel R. Rotello V. M. (2021). Nanomaterial-based therapeutics for antibiotic-resistant bacterial infections. Nat. Rev. Microbiol. 19 (1), 23–36. doi: 10.1038/s41579-020-0420-1 32814862 PMC8559572

[B94] Mala R. Aglin A. A. Celsia A. S. R. Geerthika S. Kiruthika N. VazagaPriya C.. (2017). Foley catheters functionalised with a synergistic combination of antibiotics and silver nanoparticles resist biofilm formation. IET Nanobiotechnology 11 (5), 612–620. doi: 10.1049/iet-nbt.2016.0148 28745297 PMC8676061

[B95] Maszewska A. Zygmunt M. Grzejdziak I. Różalski A. (2018). Use of polyvalent bacteriophages to combat biofilm of *Proteus mirabilis* causing catheter-associated urinary tract infections. J. Appl. Microbiol. 125 (5), 1253–1265. doi: 10.1111/jam.14026 29924909

[B96] Matsumura Y. Yoshikata K. Kunisaki S.-i. Tsuchido T. (2003). Mode of bactericidal action of silver zeolite and its comparison with that of silver nitrate. Appl. Environ. Microbiol. 69 (7), 4278–4281. doi: 10.1128/AEM.69.7.4278-4281.2003 12839814 PMC165194

[B97] Milo S. Hathaway H. Nzakizwanayo J. Alves D. R. Esteban P. Pérez Jones B. V.. (2017). Prevention of encrustation and blockage of urinary catheters by proteus mirabilis *via* PH-triggered release of bacteriophage. J. Materials Chem. B 5 (27), 5403–5411. doi: 10.1039/C7TB01302G 32264080

[B98] Mirzaei A. Wagemans J. Esfahani B. N. Lavigne R. Moghim S. (2022). A phage cocktail to control surface colonization by proteus mirabilis in catheter-associated urinary tract infections. Microbiol. Spectr. 10 (5), e02092-22. doi: 10.1128/spectrum.02092-22 36194151 PMC9602741

[B99] Mishra B. Basu A. Chua R. R. Y. Saravanan R. Tambyah P. A. Ho B.. (2014). Site Specific Immobilization of a Potent Antimicrobial Peptide onto Silicone Catheters: Evaluation against Urinary Tract Infection Pathogens. J. Materials Chem. B 2 (12), 1706. doi: 10.1039/c3tb21300e 32261400

[B100] Murray B. O. Flores C. Williams C. Flusberg D. A. Marr E. E. Kwiatkowska K. M.. (2021). Recurrent urinary tract infection: A mystery in search of better model systems. Front. Cell. Infection Microbiol. 11. doi: 10.3389/fcimb.2021.691210 PMC8188986 34123879

[B101] Murthy S. K. (2007). Nanoparticles in modern medicine: state of the art and future challenges. Int. J. Nanomedicine 2 (2), 129–141. 17722542 PMC2673971

[B102] Murugayah S. A. Gerth M. L. (2019). Engineering quorum quenching enzymes: progress and perspectives. Biochem. Soc. Trans. 47 (3), 793–800. doi: 10.1042/BST20180165 31064863 PMC6599154

[B103] Noimark S. Dunnill C. W. Kay C. W.M. Perni S. Prokopovich P. Ismail S.. (2012). Incorporation of methylene blue and nanogold into polyvinyl chloride catheters; a new approach for light-activated disinfection of surfaces. J. Materials Chem. 22 (30), 15388. doi: 10.1039/c2jm31987j

[B104] Nowatzki P. J. Koepsel R. R. Stoodley P. Min Ke Harper A. Murata H.. (2012). Salicylic acid-releasing polyurethane acrylate polymers as anti-biofilm urological catheter coatings. Acta Biomaterialia 8 (5), 1869–1880. doi: 10.1016/j.actbio.2012.01.032 22342353

[B105] Pal G. Rai P. Pandey A. (2019). “Green synthesis of nanoparticles: A greener approach for a cleaner future,” in Green synthesis, characterization and applications of nanoparticles (Elsevier Amsterdam, Netherlands), 1–26.

[B106] Paralikar P. Ingle A. P. Tiwari V. Golinska P. Dahm H. Rai M. (2019). Evaluation of antibacterial efficacy of sulfur nanoparticles alone and in combination with antibiotics against multidrug-resistant uropathogenic bacteria. J. Environ. Sci. Health Part A 54 (5), 381–390. doi: 10.1080/10934529.2018.1558892 30912480

[B107] Park J. H. Cho Y. W. Cho Y.-H. Choi J. M. Shin H. J. Bae Y. H.. (2003). Norfloxacin-releasing urethral catheter for long-term catheterization. J. Biomaterials Science Polymer Edition 14 (9), 951–962. doi: 10.1163/156856203322381438 14661872

[B108] Phuengkham H. Nasongkla N. (2015). Development of antibacterial coating on silicone surface *via* chlorhexidine-loaded nanospheres. J. Materials Science: Materials Med. 26 (2), 78. doi: 10.1007/s10856-015-5418-2 25631275

[B109] Pickard R. Lam T. MacLennan G. Starr K. Kilonzo M. McPherson G.. (2012). Types of urethral catheter for reducing symptomatic urinary tract infections in hospitalised adults requiring short-term catheterisation: multicentre randomised controlled trial and economic evaluation of antimicrobial- and antiseptic-impregnated urethral catheters (the CATHETER trial). Health Technol. Assess. 16 (47). doi: 10.3310/hta16470 23199586

[B110] Potugari B. R. Umukoro P. E. Vedre J. G. (2020). Multimodal intervention approach reduces catheter-associated urinary tract infections in a rural tertiary care center. Clin. Med. Res. 18 (4), 140–144. doi: 10.3121/cmr.2020.1533 32340983 PMC7735443

[B111] Prateeksha P. Bajpai R. Rao C. V. Upreti D. K. Barik S. K. Singh B. N. (2021). Chrysophanol-functionalized silver nanoparticles for anti-adhesive and anti-biofouling coatings to prevent urinary catheter-associated infections. ACS Appl. Nano Materials 4 (2), 1512–1528. doi: 10.1021/acsanm.0c03029

[B112] Pugach J. L. Ditizio V. Mittelman M. W. Bruce A. W. DiCosmo F. Khoury A. E. (1999). ANTIBIOTIC HYDROGEL COATED FOLEY CATHETERS FOR PREVENTION OF URINARY TRACT INFECTION IN A RABBIT MODEL. J. Urol. 162 (3 Part 1), 883–887. doi: 10.1097/00005392-199909010-00084 10458402

[B113] Rafienia M. Zarinmehr B. Poursamar S. A. Bonakdar S. Ghavami M. Janmaleki M. (2013). Coated urinary catheter by PEG/PVA/gentamicin with drug delivery capability against hospital infection. Iranian Polymer J. 22 (2), 75–83. doi: 10.1007/s13726-012-0105-3

[B114] Rahuman H. B. H. Dhandapani R. Palanivel V. Thangavelu S. Paramasivam R. Muthupandian S. (2021). Bioengineered phytomolecules-capped silver nanoparticles using carissa carandas leaf extract to embed on to urinary catheter to combat UTI pathogens. PloS One 16 (9), e0256748. doi: 10.1371/journal.pone.0256748 34473763 PMC8412375

[B115] Ramadan R. Omar N. Dawaba M. Moemen D. (2021). Bacterial biofilm dependent catheter associated urinary tract infections: characterization, antibiotic resistance pattern and risk factors. Egyptian J. Basic Appl. Sci. 8 (1), 64–74. doi: 10.1080/2314808X.2021.1905464

[B116] Ramasamy M. Lee J. (2016). Recent nanotechnology approaches for prevention and treatment of biofilm-associated infections on medical devices. BioMed. Res. Int. 2016, 1–17. doi: 10.1155/2016/1851242 PMC5107826 27872845

[B117] Reddy S. T. Chung K. K. McDaniel C. J. Darouiche R. O. Landman J. Brennan A. B. (2011). Micropatterned surfaces for reducing the risk of catheter-associated urinary tract infection: an in vitro study on the effect of sharklet micropatterned surfaces to inhibit bacterial colonization and migration of uropathogenic *Escherichia coli* . J. Endourology 25 (9), 1547–1552. doi: 10.1089/end.2010.0611 PMC3168968 21819223

[B118] Regev-Shoshani G. Ko M. Miller C. Av-Gay Y. (2010). Slow release of nitric oxide from charged catheters and its effect on biofilm formation by *Escherichia coli* . Antimicrobial Agents Chemotherapy 54 (1), 273–279. doi: 10.1128/AAC.00511-09 19884372 PMC2798533

[B119] Rémy B. Mion S. Plener L. Elias M. Chabrière E. Daudé D. (2018). Interference in bacterial quorum sensing: A biopharmaceutical perspective. Front. Pharmacol. 9. doi: 10.3389/fphar.2018.00203 PMC5845960 29563876

[B120] Rémy B. Plener L. Poirier L. Elias M. Daudé D. Chabrière E. (2016). Harnessing hyperthermostable lactonase from sulfolobus solfataricus for biotechnological applications. Sci. Rep. 6 (1), 37780. doi: 10.1038/srep37780 27876889 PMC5120315

[B121] Römling U. Balsalobre C. (2012). Biofilm infections, their resilience to therapy and innovative treatment strategies. J. Internal Med. 272 (6), 541–561. doi: 10.1111/joim.12004 23025745

[B122] Roshni A. Anne M. Venkitanaray K. (2013). “Role of bacterial biofilms in catheter-associated urinary tract infections (CAUTI) and strategies for their control,” in Recent Advances in the Field of Urinary Tract Infections (InTech). 10, 1–32.

[B123] Rtimi S. Sanjines R. Pulgarin C. Kiwi J. (2016). Quasi-instantaneous bacterial inactivation on cu–ag nanoparticulate 3D catheters in the dark and under light: mechanism and dynamics. ACS Appl. Materials Interfaces 8 (1), 47–55. doi: 10.1021/acsami.5b09730 26699928

[B124] Rubini D. Banu S. F. Subramani P. Narayanan Vedha Hari B. Gowrishankar S. Pandian S. K.. (2019). Extracted chitosan disrupts quorum sensing mediated virulence factors in urinary tract infection causing pathogens. Pathog. Dis. 77 (1), ftz009. doi: 10.1093/femspd/ftz009 30801640

[B125] Rubini D. Narayanan Vedha Hari B. Nithyanand P. (2021). Chitosan coated catheters alleviates mixed species biofilms of Staphylococcus epidermidis and candida albicans. Carbohydr. Polymers 252, 117192. doi: 10.1016/j.carbpol.2020.117192 33183634

[B126] Saini H. Chhibber S. Harjai K. (2016). Antimicrobial and antifouling efficacy of urinary catheters impregnated with a combination of macrolide and fluoroquinolone antibiotics against *Pseudomonas aeruginosa* . Biofouling 32 (5), 511–522. doi: 10.1080/08927014.2016.1155564 26982572

[B127] Saini H. Vadekeetil A. Chhibber S. Harjai K. (2017). Azithromycin-ciprofloxacin-impregnated urinary catheters avert bacterial colonization, biofilm formation, and inflammation in a murine model of foreign-body-associated urinary tract infections caused by pseudomonas aeruginosa. Antimicrobial Agents Chemotherapy 61 (3), 10–1128. doi: 10.1128/AAC.01906-16 PMC5328564 28031194

[B128] Sánchez SofíaV. Navarro Nicolás Catalán-Figueroa J. Morales J. O. (2021). Nanoparticles as potential novel therapies for urinary tract infections. Front. Cell. Infection Microbiol. 11. doi: 10.3389/fcimb.2021.656496 PMC8089393 33954121

[B129] Sarigul N. Korkmaz F. Kurultak İlhan (2019). A new artificial urine protocol to better imitate human urine. Sci. Rep. 9 (1), 20159. doi: 10.1038/s41598-019-56693-4 31882896 PMC6934465

[B130] Schairer D. O. Chouake J. S. Nosanchuk J. D. Friedman A. J. (2012). The potential of nitric oxide releasing therapies as antimicrobial agents. Virulence 3 (3), 271–279. doi: 10.4161/viru.20328 22546899 PMC3442839

[B131] Shalom Y. Perelshtein I. Perkas N. Gedanken A. Banin E. (2017). Catheters coated with zn-doped cuO nanoparticles delay the onset of catheter-associated urinary tract infections. Nano Res. 10 (2), 520–533. doi: 10.1007/s12274-016-1310-8

[B132] Siddiq D. M. Darouiche R. O. (2012). New strategies to prevent catheter-associated urinary tract infections. Nat. Rev. Urol. 9 (6), 305–314. doi: 10.1038/nrurol.2012.68 22508462

[B133] Sim W. Barnard R. T. Blaskovich M. A. T. Ziora Z. M. (2018). Antimicrobial silver in medicinal and consumer applications: a patent review of the past decade (2007–2017). Antibiotics 7, 93. doi: 10.3390/antibiotics7040093 30373130 PMC6315945

[B134] Singha P. Locklin J. Handa H. (2017). A review of the recent advances in antimicrobial coatings for urinary catheters. Acta Biomaterialia 50, 20–40. doi: 10.1016/j.actbio.2016.11.070 27916738 PMC5316300

[B135] Soto S. M. (2014). Importance of biofilms in urinary tract infections: new therapeutic approaches. Adv. Biol. 2014, 1–13. doi: 10.1155/2014/543974

[B136] Stensballe J. (2007). Infection risk with nitrofurazone-impregnated urinary catheters in trauma patients. Ann. Internal Med. 147 (5), 285. doi: 10.7326/0003-4819-147-5-200709040-00002 17785483

[B137] Stewart P. S. William Costerton J. (2001). Antibiotic resistance of bacteria in biofilms. Lancet 358 (9276), 135–138. doi: 10.1016/S0140-6736(01)05321-1 11463434

[B138] Stickler D. J. (2014). Clinical complications of urinary catheters caused by crystalline biofilms: something needs to be done. J. Internal Med. 276 (2), 120–129. doi: 10.1111/joim.12220 24635559

[B139] Syed M. A. Manzoor U. Shah I. Habib Ali Bukhari S. (2010). Antibacterial effects of tungsten nanoparticles on the escherichia coli strains isolated from catheterized urinary tract infection (UTI) cases and staphylococcus aureus. New Microbiologica 33 (4), 329–335. 21213591

[B140] Tailly T. MacPhee R. A. Cadieux P. Burton J. P. Dalsin J. Wattengel C.. (2021). Evaluation of polyethylene glycol-based antimicrobial coatings on urinary catheters in the prevention of *Escherichia coli* infections in a rabbit model. J. Endourology 35 (1), 116–121. doi: 10.1089/end.2020.0186 PMC7876351 32689838

[B141] Tarawneh O. Abu Mahfouz H. Hamadneh L. Deeb A. A. Al-Sheikh I. Alwahsh W.. (2022). Assessment of persistent antimicrobial and anti-biofilm activity of p-HEMA hydrogel loaded with rifampicin and cefixime. Sci. Rep. 12 (1), 3900. doi: 10.1038/s41598-022-07953-3 35273262 PMC8913786

[B142] Tenke P. Kovacs B. Bjerklund Johansen T. E. Matsumoto T. Tambyah P. A. Naber K. G. (2008). European and asian guidelines on management and prevention of catheter-associated urinary tract infections. Int. J. Antimicrobial Agents 31, 68–78. doi: 10.1016/j.ijantimicag.2007.07.033 18006279

[B143] Tenke P. Köves Béla Nagy Károly Hultgren S. J. Mendling W. Wullt Björn. (2012). Update on biofilm infections in the urinary tract. World J. Urol. 30 (1), 51–57. doi: 10.1007/s00345-011-0689-9 21590469 PMC4629855

[B144] Thallinger B. Brandauer M. Burger P. Sygmund C. Ludwig R. Ivanova K.. (2016). Cellobiose dehydrogenase functionalized urinary catheter as novel antibiofilm system. J. Biomed. Materials Res. Part B: Appl. Biomaterials 104 (7), 1448–1456. doi: 10.1002/jbm.b.33491 26251187

[B145] Thomas R. Soumya K. R. Mathew J. Radhakrishnan E. K. (2015). Inhibitory effect of silver nanoparticle fabricated urinary catheter on colonization efficiency of coagulase negative staphylococci. J. Photochem. Photobiol. B: Biol. 149, 68–77. doi: 10.1016/j.jphotobiol.2015.04.034 26048526

[B146] Thomé I. P. S. Dagostin V. S. Piletti R. Pich C. T. Riella H. G. Angioletto E.. (2012). Bactericidal low density polyethylene (LDPE) urinary catheters: microbiological characterization and effectiveness. Materials Sci. Engineering: C 32 (2), 263–268. doi: 10.1016/j.msec.2011.10.027

[B147] Thompson V. C. Adamson P. J. Dilag J. Uswatte Liyanage D. B. U. Srikantharajah K. Blok A.. (2016). Biocompatible anti-microbial coatings for urinary catheters. RSC Adv. 6 (58), 53303–53309. doi: 10.1039/C6RA07678E

[B148] Trautner B. W. Hull R. A. Darouiche R. O. (2003). Escherichia coli 83972 inhibits catheter adherence by a broad spectrum of uropathogens. Urology 61 (5), 1059–1062. doi: 10.1016/S0090-4295(02)02555-4 12736047 PMC2963591

[B149] Vaterrodt A. Thallinger B. Daumann K. Koch D. Guebitz G. M. Ulbricht M. (2016). Antifouling and antibacterial multifunctional polyzwitterion/enzyme coating on silicone catheter material prepared by electrostatic layer-by-layer assembly. Langmuir 32 (5), 1347–1359. doi: 10.1021/acs.langmuir.5b04303 26766428

[B150] Vidyavathi M. Srividya G. (2018). A REVIEW ON CIPROFLOXACIN: DOSAGE FORM PERSPECTIVE. Int. J. Appl. Pharmaceutics 10 (4), 6. doi: 10.22159/ijap.2018v10i4.25315

[B151] Wang X.-w. Wang J. Yu Y. Yu Lu Wang Y.-x. Ren K.-f.. (2022). A polyzwitterion-based antifouling and flexible bilayer hydrogel coating. Composites Part B: Eng. 244, 110164. doi: 10.1016/j.compositesb.2022.110164

[B152] Weatherly L. M. Gosse J. A. (2017). Triclosan exposure, transformation, and human health effects. J. Toxicol. Environ. Health Part B 20 (8), 447–469. doi: 10.1080/10937404.2017.1399306 PMC6126357 29182464

[B153] Werneburg G. T. (2022). Catheter-associated urinary tract infections: current challenges and future prospects. Res. Rep. Urol. 14, 109–133. doi: 10.2147/RRU.S273663 35402319 PMC8992741

[B154] Xiong G.-B. Xie S.-H. Liu A.-B. (2021). *In* v*itro* dynamic bladder models for studying urinary tract infections: A narrative review. Ann. Palliative Med. 10 (4), 4830–4839. doi: 10.21037/apm-20-2061 33691461

[B155] Yang K. Kim K. Lee E. A. Liu S. S. Kabli S. Alsudir S. A.. (2019). Robust low friction antibiotic coating of urethral catheters using a catechol-functionalized polymeric hydrogel film. Front. Materials 6. doi: 10.3389/fmats.2019.00274

[B156] Yassin M. A. Elkhooly T. A. Elsherbiny S. M. Reicha F. M. Shokeir A. A. (2019). Facile coating of urinary catheter with bio–inspired antibacterial coating. Heliyon 5 (12), e02986. doi: 10.1016/j.heliyon.2019.e02986 31886428 PMC6921108

[B157] Yu K. Lo J. C.Y. Yan M. Yang X. Brooks D. E. Hancock R. E.W.. (2017). Anti-adhesive antimicrobial peptide coating prevents catheter associated infection in a mouse urinary infection model. Biomaterials 116, 69–81. doi: 10.1016/j.biomaterials.2016.11.047 27914268

[B158] Yueh M.-F. Tukey R. H. (2016). Triclosan: A widespread environmental toxicant with many biological effects. Annu. Rev. Pharmacol. Toxicol. 56 (1), 251–272. doi: 10.1146/annurev-pharmtox-010715-103417 26738475 PMC4774862

[B159] Zhang D. Guolong Z. Hayden M. S. Greenblatt M. B. Bussey C. Flavell R. A.. (2004). A toll-like receptor that prevents infection by uropathogenic bacteria. Science 303 (5663), 1522–1526. doi: 10.1126/science.1094351 15001781

[B160] Zhang S. Liang X. Gadd G. M. Zhao Qi (2020). Superhydrophobic coatings for urinary catheters to delay bacterial biofilm formation and catheter-associated urinary tract infection. ACS Appl. Bio Materials 3 (1), 282–291. doi: 10.1021/acsabm.9b00814 35019444

[B161] Zhang S. Liang X. Gadd G. M. Zhao Qi (2021). Marine microbial-derived antibiotics and biosurfactants as potential new agents against catheter-associated urinary tract infections. Mar. Drugs 19 (5), 255. doi: 10.3390/md19050255 33946845 PMC8145997

[B162] Zhang S. Wang L. Liang X. Vorstius J. Keatch R. Corner G.. (2019). Enhanced antibacterial and antiadhesive activities of silver-PTFE nanocomposite coating for urinary catheters. ACS Biomaterials Sci. Eng. 5 (6), 2804–2814. doi: 10.1021/acsbiomaterials.9b00071 33405585

